# Pooled screening of CAR T cells identifies diverse immune signaling domains for next-generation immunotherapies

**DOI:** 10.1126/scitranslmed.abm1463

**Published:** 2022-11-09

**Authors:** Daniel B. Goodman, Camillia S. Azimi, Kendall Kearns, Alexis Talbot, Kiavash Garakani, Julie Garcia, Nisarg Patel, Byungjin Hwang, David Lee, Emily Park, Vivasvan S. Vykunta, Brian R. Shy, Chun Jimmie Ye, Justin Eyquem, Alexander Marson, Jeffrey A. Bluestone, Kole T. Roybal

**Affiliations:** 1 Department of Microbiology and Immunology, University of California, San Francisco; San Francisco, California, 94143, USA.; 2 Parker Institute for Cancer Immunotherapy; San Francisco, California, 94143, USA.; 3 Helen Diller Family Comprehensive Cancer Center, University of California, San Francisco; San Francisco, California, 94158, USA.; 4 Chan Zuckerberg Biohub; San Francisco, California, 94158, USA.; 5 Gladstone UCSF Institute for Genetic Immunology; San Francisco, CA, 94107, USA.; 6 UCSF Cell Design Institute; San Francisco, California, 94158, USA.; 7 Department of Oral and Maxillofacial Surgery, University of California, San Francisco; San Francisco, CA, USA.; 8 Bakar Computational Health Sciences Institute, University of California, San Francisco; San Francisco, CA, USA.; 9 School of Medicine, University of California, San Francisco; San Francisco, CA, USA.; 10 Institute for Human Genetics (IHG), University of California, San Francisco; San Francisco, California, USA.; 11 Department of Epidemiology and Biostatistics, San Francisco; San Francisco, CA 94143, USA.; 12 Innovative Genomics Institute, University of California, Berkeley; Berkeley, CA 94720, USA.; 13 Diabetes Center, University of California, San Francisco; San Francisco, CA 94143, USA.; 14 Sonoma Biotherapeutics; South San Francisco, CA, USA.; 15 Department of Medicine, University of California, San Francisco; San Francisco, California, 94143, USA.; 16 INSERM U976, Saint Louis Research Institute, Paris City University, Paris, France.

## Abstract

Chimeric antigen receptors (CARs) repurpose natural signaling components to retarget T cells to refractory cancers but have shown limited efficacy in persistent, recurrent malignancies. Here, we introduce “CAR Pooling”, a multiplexed approach to rapidly identify CAR designs with clinical potential. Forty CARs with signaling domains derived from a range of immune cell lineages were evaluated in pooled assays for their ability to stimulate critical T cell effector functions during repetitive stimulation that mimics long-term tumor antigen exposure. Several domains were identified from the tumor necrosis factor (TNF) receptor family that have been primarily associated with B cells. CD40 enhanced proliferation, whereas B-cell activating factor receptor (BAFF-R) and transmembrane activator and CAML interactor (TACI) promoted cytotoxicity. These functions were enhanced relative to clinical benchmarks after prolonged antigen stimulation, and CAR T cells signaling through these domains fell into distinct states of memory, cytotoxicity, and metabolism. BAFF-R CAR T cells were enriched for a highly cytotoxic transcriptional signature previously associated with positive clinical outcomes. Additionally, we observed that replacing the 4–1BB intracellular signaling domain with the BAFF-R signaling domain in a clinically validated B-cell maturation antigen (BCMA)-specific CAR resulted in enhanced activity in a xenotransplant model of multiple myeloma. Together, these results show that “CAR Pooling” is a general approach for rapid exploration of CAR architecture and activity to improve the efficacy of CAR T cell therapies.

## One Sentence Summary

CAR Pooling is a high-throughput approach to screening and testing CAR designs with the potential to enhance engineered T cell therapies for cancer.

## INTRODUCTION

Adoptive cell therapy using engineered chimeric antigen receptor (CAR) T cells has revolutionized the treatment of B-cell leukemias and lymphomas(1, 2). CAR T cells currently in the clinic use either a 4-1BB or CD28 intracellular costimulatory domain, which come from natural, well-studied T cell costimulatory receptors. Costimulation is a critical component of immune activation, and CARs lacking the “signal 2” from a costimulatory domain quickly become anergic upon stimulation(3). CARs containing 4-1BB and CD28 intracellular domains are used in second generation CAR T cells, which elicit more robust and sustained T cell activation than the original CD3ζ-only CARs. Although both CD28- and 4-1BB-CAR T cells are effective therapeutics, there are substantial differences in their synapse development, cytotoxicity, metabolic state, and clinical performance(4–7). Additionally, preclinical and clinical studies show that T cells expressing CD28 CARs are initially faster to proliferate and kill tumor cells, but suffer from reduced long-term engraftment and heightened exhaustion after prolonged activation(8–12).

There is considerable diversity in costimulatory domains, and evidence for both quantitative and qualitative differences in costimulatory signaling in the context of a CAR(7). 4-1BB and CD28 utilize two separate signaling pathways [tumor necrosis factor (TNF) receptor associated factor (TRAF) and phosphatidylinositol-3-kinase (PI3K)/lymphocyte-specific protein tyrosine kinase (Lck)/growth factor receptor-bound protein 2 (Grb2), respectively], however these pathways converge upon conserved signaling intermediates, suggesting that costimulatory domains from other immune cells may also be able to signal in T cells(13–15). Other studies have individually characterized additional T cell costimulatory domains within CARs or searched for mutant domains with enhanced properties(16–20). However, the scale of these searches has been limited to selected domains, focused on receptors with known functions in T cells.

Pooled screens are a powerful tool for probing T cell biology, including using clustered regularly interspaced short palindromic repeats (CRISPR) knockouts and switch receptors(21, 22), but have only recently been applied to CAR engineering. Pooled assays offer increased throughput and direct comparison of cells from the same blood donor tested in identical conditions. Screening of large numbers of domains to assess their effects on multiple cell-intrinsic T cell phenotypes would help identify optimal CAR designs for clinical applications. Although pooled measurement can be applied to many aspects of CAR architecture, signaling domains - which are small, have minimal secondary structure, consist of short and modular signaling motifs(23) - lend themselves to pooled characterization using large synthetic DNA libraries(24). Signaling domains identified from these screens can then be more deeply characterized for potential clinical translation.

The lack of persistence and long-term efficacy in patients is a central problem for current CAR T therapies, in both solid cancers and hematological tumors such as multiple myeloma(25–27). To answer this clinical need, we assembled a signaling domain library consisting of both inhibitory and stimulatory domains from a range of innate and adaptive immune cells to assess their propensity to resist exhaustion within the CAR architecture(3). We then performed a suite of pooled assays in primary human CD4 or CD8 T cells containing this CAR library, using a repetitive stimulation assay we developed to mimic the protracted stress and exhaustion of chronic antigen exposure on T cells in difficult-to-eliminate tumors. The dataset produced from these assays represents a systematic survey of the CAR T cell costimulation landscape. We identified a set of potent costimulatory domains from the TNF receptor family – CD40, B-cell activating factor receptor (BAFF-R), and transmembrane activator and CAML interactor (TACI) – that drove T cells to exhibit enhanced proliferation and cytotoxicity in vitro, despite the domains being primarily associated with the B cell lineage. We also identified killer cell lectin like receptor G1 (KLRG1), an inhibitory domain, which silenced CD3ζ activation and kept CAR T cells in a naive transcriptional state. Additionally, single-cell RNA and surface protein expression profiles among these candidates showed that CAR T cells using the signaling domain of BAFF-R were enriched for a highly cytotoxic transcriptional signature, which has been associated with enhanced CAR T engraftment and improved response against melanoma in clinical studies(28, 29). BAFF-R-based CAR T cells, as compared to benchmark 4-1BB- and CD28-based CAR T cells, exhibited equivalent efficacy in a mouse model of mesothelioma and superior efficacy in a model of multiple myeloma.

## RESULTS

### CAR Pooling allows for screening of a pooled library of CARs with diverse costimulatory domains.

**Fig. 1.**
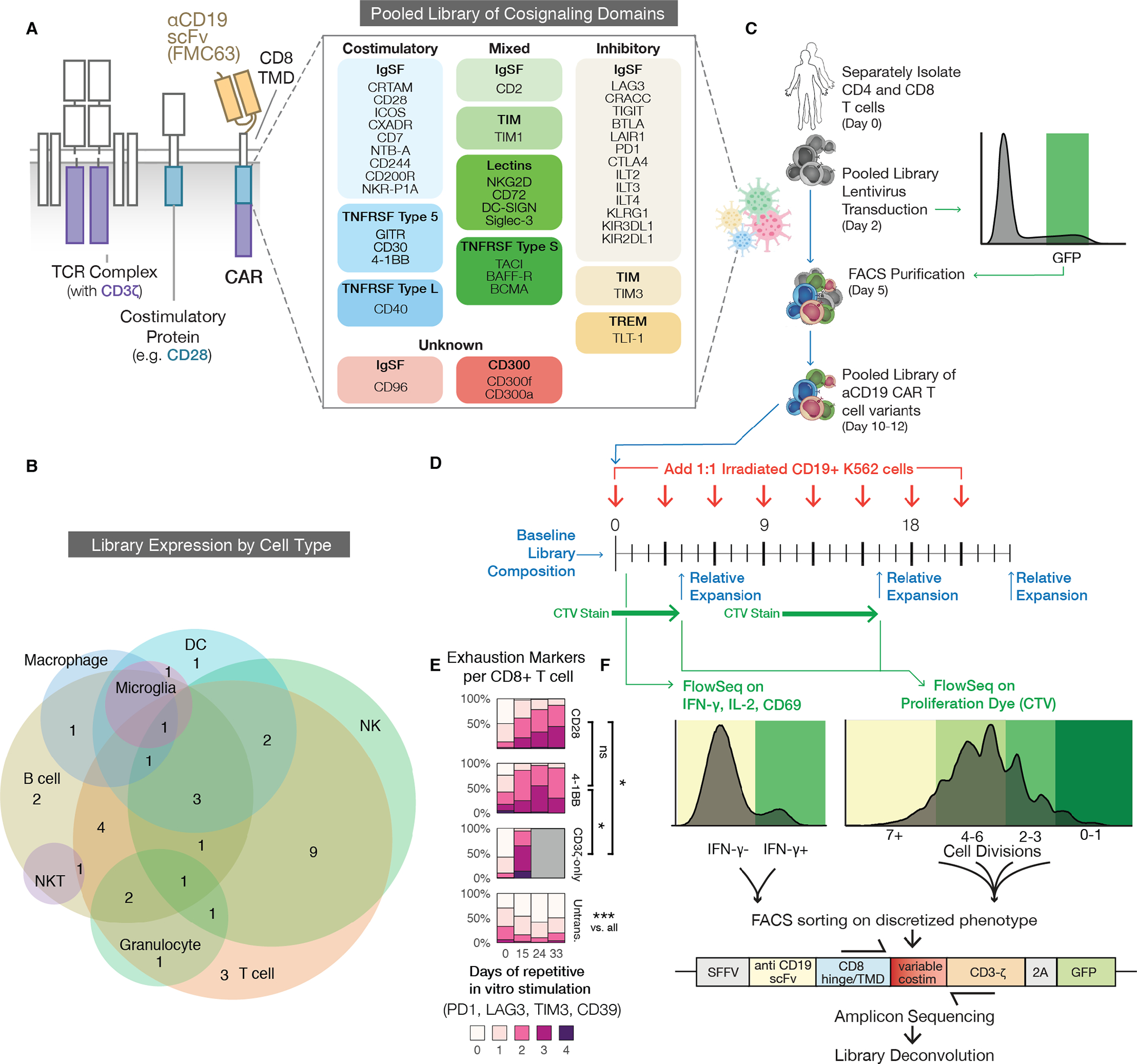
Generation and screening of a pooled library of CARs with diverse signaling domains. **(A)** Our CARs combine an αCD19 ScFv (FMC63), a CD8 hinge and transmembrane domain (TMD), an intracellular signaling domain, and a CD3ζ domain (left). Forty domains from across the human proteome were codon-optimized, synthesized, pooled into a plasmid library, and packaged to generate lentivirus (right). These spanned protein families such as immunoglobulin superfamily (IgSF), T cell/transmembrane, immunoglobulin, and mucin (TIM), TNF receptor super family (TNFRSF), and triggering receptors expressed on myeloid cells (TREM). **(B)** A Venn diagram showing natural expression of library members across immune cell types. Expression patterns are listed in table S1. NK, natural killer cell; NKT, natural killer T cell; DC, dendritic cell. **(C)** Primary human CD4 and CD8 T cells were separately isolated from PBMCs for two human donors and lentivirally transduced with the library. The cells were FACS purified using T2A-GFP fluorescence within one log of mean expression to reduce variability. **(D)** The pooled library was repeatedly stimulated 1:1 with CD19+ or CD19- irradiated K562 tumor cells to quantify antigen specific activation (CD69), cytokine production (IFN-γ, IL-2), and proliferation (CTV: CellTrace Violet). The library was also sequenced at the specified timepoints to measure relative expansion of individual constructs. **(E)** Percentage of CD8+ T cells expressing different numbers of exhaustion markers (PD1, TIM3, LAG3, CD39) after a repeat stimulation assay with CD19+ irradiated K562 tumor cells. T cells expressing CD28, 4-1BB, or CD3ζ-only CARs are compared to untransduced (Untrans.) cells. Grey boxes correspond to timepoints in which no live cells remained. Significance was assessed in CD8 T cells for 2 donors using a Repeated Measures ANOVA model. FDR-corrected *p* < 0.05:*, *p*<0.001:***; ns, not significant. The second donor and data for CD4+ T cells are shown in fig. S1 A and B. **(F)** We used FlowSeq, a FACS and next generation sequencing (NGS)-based pooled quantification workflow, to quantify enrichment by sorting the library into bins of fluorescent signal corresponding to a functional readout, as shown by the colored histograms. We then amplified the costimulatory domain by genomic DNA extraction and PCR, and performed amplicon sequencing on each bin to estimate the phenotype for each library member.

Innate and adaptive immune cells use a set of specialized receptors to sense their extracellular environment and elicit critical cellular functions. To transduce these signals, these receptors often use modular linear signaling motifs within their intracellular domains, which bind to downstream signaling proteins. These motifs and signaling partners are often highly conserved across the immune system(3, 23), and we hypothesized that some of these unexplored signaling domains and motifs could engage distinct and beneficial T cell signaling in the context of a CAR. To test this, we developed a method for high-throughput, pooled screening of CAR signaling libraries within primary human T cells, which we call “CAR Pooling”. To generate the CAR Pooling library, we mined 40 costimulatory and coinhibitory receptor intracellular domains from different protein families and functional classes associated with several immune cell types, including natural killer (NK) cells, B cells, and other innate immune cells (Fig. 1A and B, table S1). We synthesized and cloned the domains into a second-generation CAR scaffold and lentivirally transduced the CAR into independent CD4 and CD8 primary human T cell cultures from matched donors (Fig. 1C). CAR-positive T cells were sorted for a defined range of CAR expression using a 2A-green fluorescent protein (GFP) marker and rested before proceeding with tumor cell stimulation (Fig. 1C).

To mimic the exhaustion conditions encountered in patients with a high tumor burden, we performed repetitive, long-term in vitro stimulations of the CAR T cells over 24 to 33 days, with CD19+ or CD19- K562 cells added 1:1 to the T cell culture every three days (Fig. 1D). K562 cells were irradiated prior to their addition to reduce their proliferative capacity and prevent rapid depletion of the media. CD28 and 4-1BB CAR T cells in this assay became increasingly exhausted (Fig. 1E, fig. S1A and B). Additionally, a CAR containing only the CD3ζ domain appeared to have an anergic phenotype by day 15 and did not survive after day 24, indicating that the effect of costimulation, which is critical to durable responses in vivo, is at least partially captured by this model(7, 30–32).

**Fig. 2.**
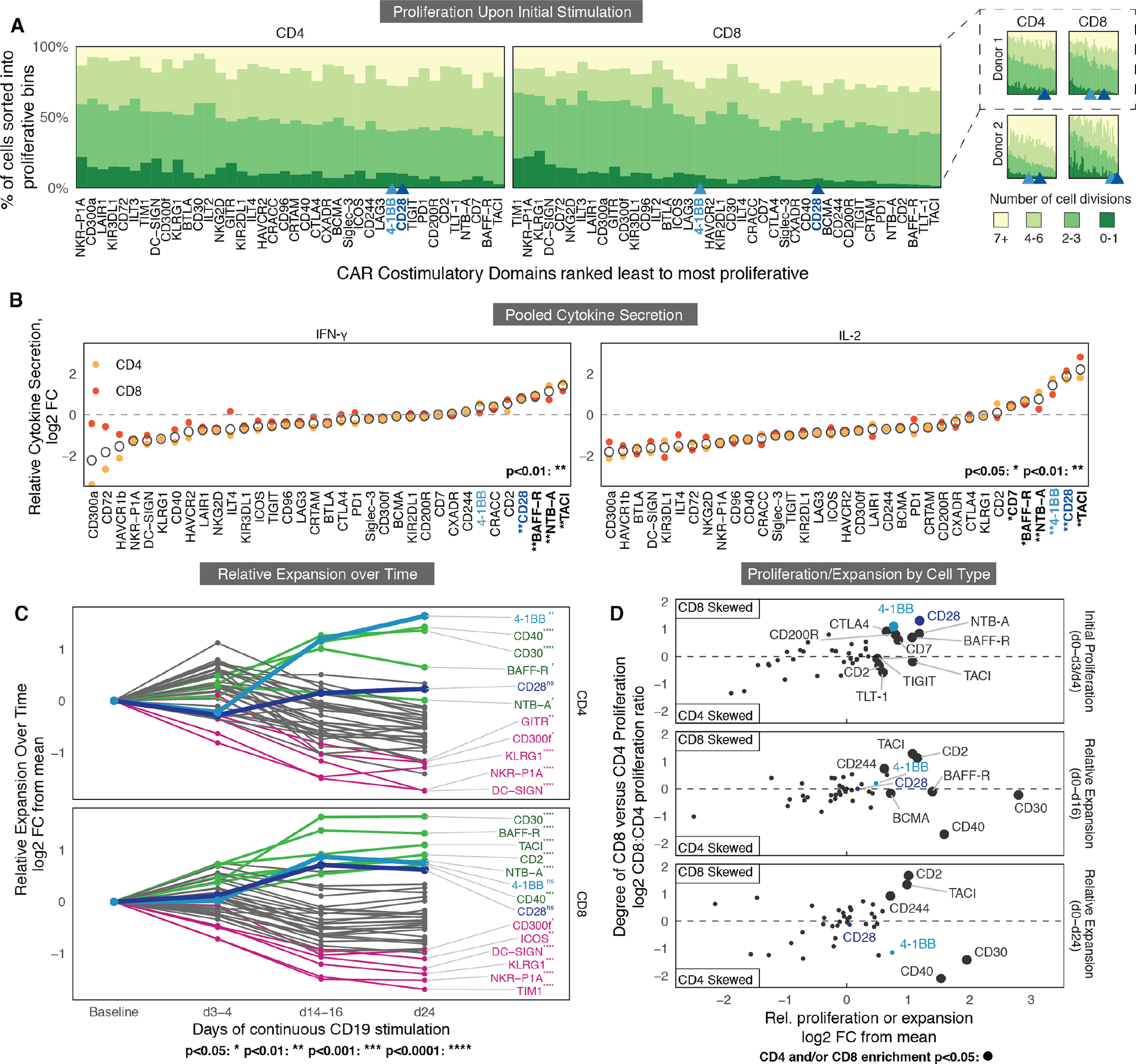
Comparison of proliferation, expansion, and cytokine secretion identifies differences in costimulatory activity at different time scales and in different T cell types. **(A)** FlowSeq measurement of proliferation are shown for CD4 (left) and CD8 (right) T cells containing different signaling domains, separately stimulated in vitro with irradiated CD19+ K562 tumor cells for 3 or 4 days (see Fig. 1D). Percentage of cells with different numbers of divisions were calculated from amplicon sequencing of sorted CTV bins. CARs are ranked from left to right by the average number of cell divisions in CD4 or CD8 cells from lowest (left) to highest (right). 4-1BB and CD28 CARs are highlighted in blue shades. On the right, two donors are shown separately along with 4-1BB and CD28 rank. **(B)** FlowSeq measurements of intracellular cytokine production are shown for CD4 (yellow) and CD8 (orange) T cells averaged across three independent donors, 18 hours after addition of CD19+ irradiated K562 cells. Mean of each domain is indicated by an open circle. Dashed lines indicate the cytokine production of the average library member, normalized for each donor and T cell subset. Significance was determined using a Wilcoxon rank-sum test. FC, fold-change. **(C)** Relative expansion over time is shown for CD4 and CD8 T cells, based on the average fold-change in library abundance from baseline before stimulation. Mean of 3 replicates from 2 donors are shown. The 6 domains with the most and least relative expansion are labeled in green and pink, respectively, with CD28 and 4-1BB labeled in blue shades. Significance is based on FDR-corrected *p* values derived from a linear mixed effects model (see Methods). **(D)** Comparison of CD4 versus CD8 proliferation and expansion after CD19+ K562 stimulation. Larger dots correspond to constructs that have significantly better performance in either CD4 T cells or CD8 T cells. The x-axis is the mean fold-change across CD4 and CD8 T cells, and the y-axis is the CD8:CD4 expansion ratio. Significance was determined using a Wald test. For all panels, FDR < 0.05:*, < 0.01:**, <0.001:***, <0.0001:****.

To efficiently characterize the CAR Pooling library across multiple assays and time points, we employed FlowSeq, a pooled flow-cytometry-based method which quantitatively measures any cell-based fluorescent readout across a genetically-diverse population of cells(33). We used FlowSeq to measure different markers of T cell function, such as activation (CD69), cytokine production [interferon (IFN)-γ and interleukin (IL)-2], and proliferation using CellTrace Violet (CTV) dye. For each pooled assay, cells were sorted and separately sequenced to compare the functional differences among the domains (Fig. 1D and F; Fig. 2A and B). This multiplexed approach allowed us to compare differential CAR T activity between CD4 and CD8 T cell types, and between early and late stages of antigen stimulation and expansion.

Despite donor variability, the domain ranking across replicates for all assays was consistent and highly correlated (Kruskal-Wallis *H* > 110; *p* < 1e-10) (fig. S1C). We found no correlation among the library domains between either initial domain abundance or length and early or late relative expansion (fig. S1D). Given these measurements, we determined that CAR Pooling is a generally reproducible and robust platform for these multiplexed assays.

### Multidimensional comparison of signaling domains across repetitive expansion identifies new potent costimulatory domains.

Antigen-induced proliferation, cytokine secretion, and activation varied across CAR T cells expressing different costimulatory domains (Fig. 2A, fig. S2A to C), and the canonical CD28 and 4-1BB domains were among those that promoted the most cytokine secretion and proliferation (average rank 4 and 5, respectively; Mann-Whitney U, *p* < 9.6e-8, across all assays). However, other domains consistently appeared among the top-performers, including BAFF-R and TACI (average ranks 1 and 2, respectively).

Antigen-independent proliferation also varied considerably among the domains. Although overall proliferation was universally lower without antigen, domains that promoted strong proliferation upon antigen-exposure also tended to exhibit enhanced proliferation in antigen-negative co-culture (Spearman’s ⍴ = 0.63–0.75, p = 1.3×10^−5^), indicating a strong correlation between antigen-dependent proliferation and increased basal proliferation (fig. S2B). Among the top-performing domains, CD28 and TACI exhibited the highest degree of non-specific proliferation, whereas CD40 had the lowest (fig. S2B). We measured IL-2 and IFN-γ production (Fig. 2B, fig. S2C) and CD69 expression (fig. S2D) in both CD4 and CD8 T cells. Ranking of CARs from least to most cytokine production in log2 fold change, IL-2 and IFN-γ secretion was similar in multiple cultures of CD4 and CD8 T cells (yellow, orange) across three human donors (Fig. 2B, fig. S2C).

The lack of long-term CAR T cell persistence is often cited as a major reason for antigen-positive relapse in patients (34). Multiple aspects of cell dynamics underlie CAR T cell persistence, including a cell’s lifespan during and after stimulation. Cell proliferation (CTV) is thus an incomplete representation of CAR T efficacy. To better capture persistence, we also measured change in the relative frequency of a domain over time, termed “relative expansion”. We compared amplicon sequencing of the library immediately before stimulation and subsequently after the first, sixth, and eighth stimulations (days 3 or 4, 14 or 16, and 24, respectively, for CD4 T cells and CD8 T cells) (Fig. 2C). Many of the domains that preferentially expanded after the first stimulation were subsequently diminished after further stimulations, indicating that initial proliferation did not correlate well with long-term proliferative capacity and persistence (fig. S2E). We also saw some domains differentially enriched in either CD4 or CD8 T cells (Fig. 2D). Most domains promoted a greater expansion overall in CD8 T cells, with the notable exceptions of CD30, CD40, and 4-1BB, where, after 24 days of stimulation, CD4 T cells expanded over two-fold more than CD8 T cells. As noted by previous studies, these results imply that using different costimulatory domains in CD4 versus CD8 T cells may improve overall CAR T therapeutic efficacy (17).

### Scoring CARs across pooled measurements identifies signaling domains with distinct stimulatory activity.

**Fig. 3.**
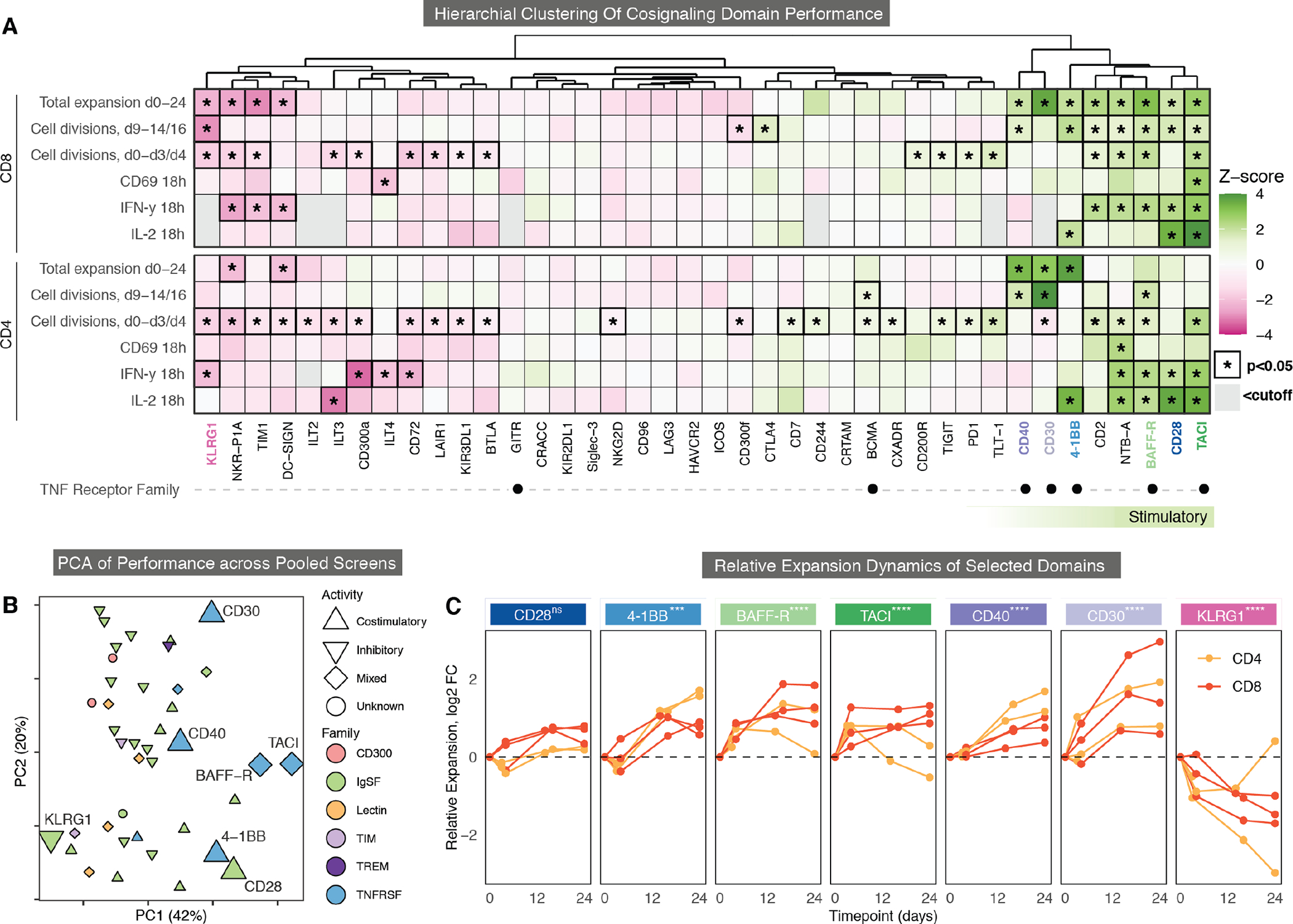
Multidimensional comparison of signaling domains across multiple weeks of chronic antigen stimulation identifies a subset with potent stimulatory activity. **(A)** Hierarchical clustering of the CAR signaling domain library in CD4 and CD8 T cells stimulated with CD19+ K562 tumor cells. All 40 domains were clustered based on their z-scores in each assay. 8 stimulatory domains were identified on the right (green bar). Gray boxes are excluded domains where < 500 cells were detected for the assay. TNF receptor family members are marked with black dots. KLRG1 (pink), CD40 (dark purple), CD30 (light purple), 4-1BB (light blue), BAFF-R (light green), CD28 (dark blue), and TACI (dark green) are highlighted. Significance is indicated by a black border and an asterisk(Wilcoxon rank-sum test, FDR < 0.05). **(B)** Principal components analysis (PCA) of pooled library screen cytokine, proliferation, expansion, and activation data is shown for CD4 T cells, CD8 T cells, CD19+ and CD19− stimulation conditions, and all donors and timepoints. Chosen CARs are larger, with shapes and color indicating known function and protein family. **(C)** Fold-change of the proportion of selected CARs within the library at each timepoint (x-axis) over 24 days of repeated stimulation with irradiated CD19+ K562 cells as compared to the average CAR in the pooled library. CARs were measured in CD4 and CD8 primary human T cells individually in 2 to 3 biological replicates. Significant p values were derived from a linear mixed effects model; FDR < 0.05:*, < 0.01:**, <0.001:***, <0.0001:****.

To summarize the relative performance of all domains over the repetitive stimulation assays, hierarchical clustering was performed (Fig. 3A). A subset of CAR T cells containing CD28, 4-1BB, and several additional domains clustered into a potent costimulatory group, demonstrating enhanced T cell functions relative to the average domain in the library (Fig. 3A). This group was highly enriched for domains belonging to the TNF receptor family. CD40 and CD30 were most similar to 4-1BB, demonstrating substantial overall expansion and late-stage proliferation in both CD4 and CD8 T cells. In contrast, BAFF-R, CD28, and TACI showed moderately enhanced expansion and late-stage proliferation but promoted substantial cytokine production, with CD28 and TACI producing high IL-2. Overall, the eight most potent CAR costimulatory domains were distributed along a spectrum of late-stage proliferators to high-cytokine producers, suggesting that there may be inherent tradeoffs between these two aspects of CAR T activity. As members of the TNF receptor family spanned the stimulatory spectrum, we highlighted BAFF-R (light green), TACI (dark green), CD40 (light purple), and CD30 (dark purple).

Some domains, such as KLRG1 and NKR-P1A, consistently demonstrated the lowest initial proliferation, activation, and cytokine production in both CD4 and CD8 T cells (Fig. 3A). These are also of interest because inhibitory signaling could be used to halt, dampen, or dynamically modulate T cell functions (35). For this reason, we added KLRG1, a potential inhibitory domain (pink), in our subsequent investigations.

To further compare overall library performance, a principal component analysis (PCA) was performed across all measurements with and without antigen stimulation (Fig. 3B, fig. S3A and B). The PCA showed that domains are spread across a diverse signaling landscape, with principal component (PC) 1 being associated with early proliferation, cytokine secretion, CD69 activation, and more tonic signaling; in contrast, PC2 was associated with long-term expansion in CD8 T cells, less cytokine secretion, less early proliferation, and relatively less tonic signaling (fig. S3A). PC2 was also correlated with domain size, suggesting that increasing the distance between the membrane and CD3ζ reduces the strength of early activation and tonic signaling (fig. S3B). This is supported by recent work showing that altering the position of CD3ζ immunoreceptor tyrosine-based activation motifs (ITAMs) modulates differentiation and memory formation(36–39). There was no discernable clustering based on cell-type specific expression or protein family structure, aside from the enrichment of TNF family members in the stimulatory group (Fig. 3B). Additionally, there were only minor differences in cell surface expression for each of the highlighted CARs measured using an N-terminal myc tag normalized for transduction through the co-expressed T2A GFP reporter (fig. S3C).

CAR T cells that used BAFF-R, TACI, CD40, or CD30 also enhanced persistence in the pooled library, demonstrated by the dynamics of their relative expansion through day 24 (Fig. 3C). In addition, CD30 and CD40 showed the most and least antigen-independent expansion, respectively (fig. S3D). Lastly, as our highlighted domains all belong to the TNF receptor family, and thus all use TRAF signaling (40), we surveyed their literature-annotated TRAF binding sites and post-translational modifications (41) to assess shared signaling characteristics (fig. S3E). They can associate with a diverse set of TRAFs, with no discernible shared motif or partner across the 5 domains (42–45).

### A subset of signaling domains differentially affects proliferation, long-term expansion, and late-stage metabolism.

**Fig. 4.**
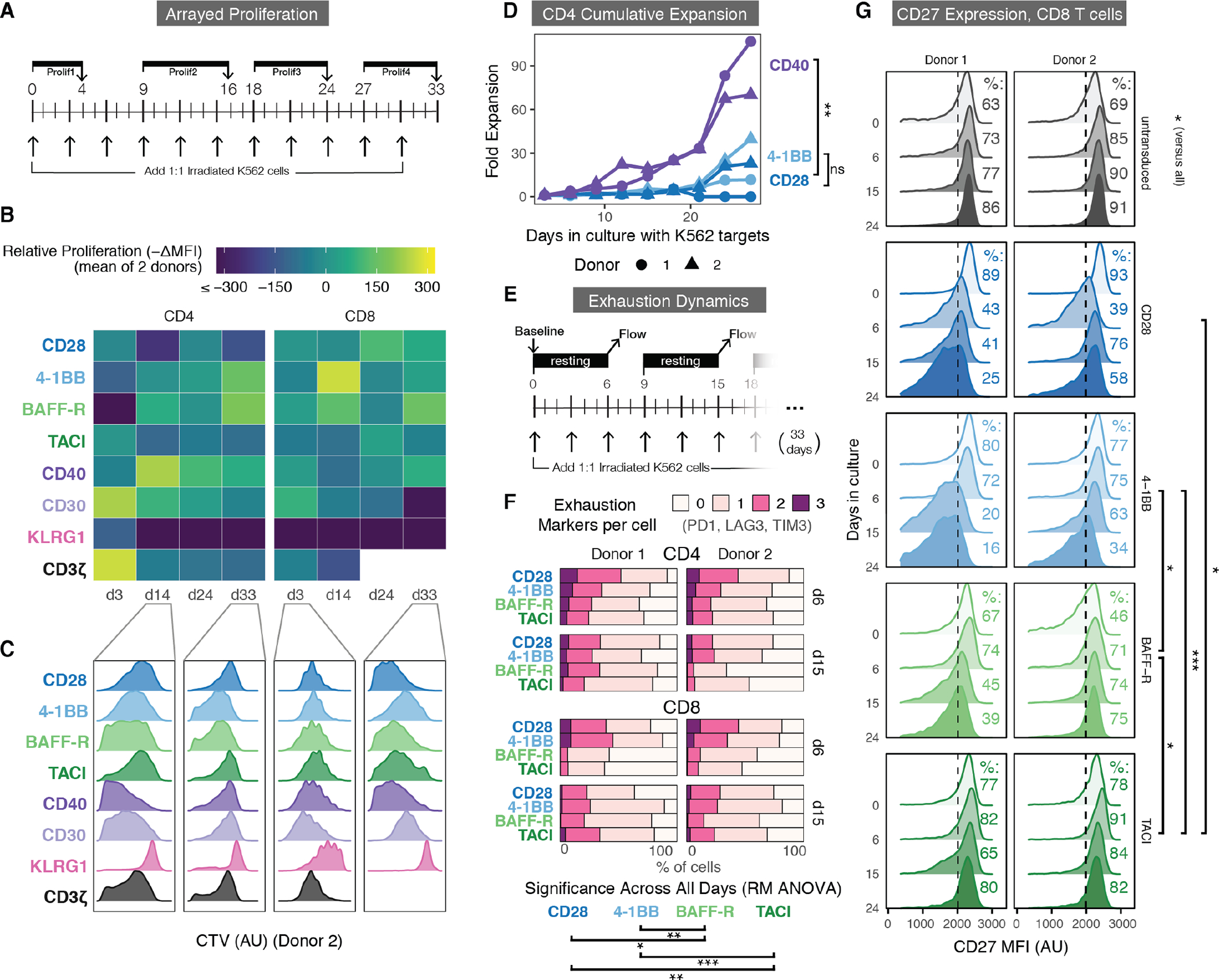
Distinct signaling domains differentially affects proliferation, long-term expansion, markers of memory, and exhaustion. **(A)** The timeline for arrayed proliferation assays in (B and C) is shown. Primary human CD4 and CD8 T cells were separately transduced with the 8 CARs in (B and C) and stimulated 1:1 with irradiated CD19+ or CD19- K562 cells every three days. Proliferation was assessed by CTV dilution every 9 days. **(B)** Relative proliferation of each CAR was quantified by the relative decrease in mean fluorescence intensity (MFI), representing the dilution of CTV dye, between two donors of CD4 or CD8 T cells. The color legend ranges from less proliferative (dark blue) to more proliferative (yellow). The x-axis indicates the day the cells were stained. White boxes are representative of CAR T cells that dropped out of culture. **(C)** Histograms of CTV staining of CD4 or CD8 CAR T cells in a representative donor on selected days are shown. Data summarized in (B). Both donors are shown in fig. S4A and B. AU, arbitrary units. **(D)** Quantification of the cumulative expansion of CD4 T cells engineered with either CD40 (purple), 4-1BB (light blue), or CD28 (dark blue) CARs and stimulated as described in (A). Co-cultures were measured every three days starting on day 3 by flow cytometry and counting beads. The y-axis measures cumulative fold-expansion every three days. (Significance was derived from a linear mixed effects model, for CD40 comparisons, all p < .001). **(E)** Cells were transduced and stimulated as described in (A). Every 9 days in culture, cells were rested for 6 days without additional stimulation and assessed for surface expression of PD1, LAG3, and TIM3. **(F)** Percentage of CAR T cells expressing 0 to 3 of the exhaustion markers PD1, TIM3, LAG3 after day 6 and day 15 as described in (E). All CARs, markers, and time points are shown in fig. S4D and E. **(G)** CD27 surface expression on CD8 CAR T cells was measured over 33 days, as in (E). Percentage of CD27-high cells is shown for each CAR and day on the right. All CARs and time points are shown in fig. S4G. Significance in (F and G) was assessed using a repeated measures (RM) ANOVA model, (FDR < 0.05:*, < 0.01:**, < 0.001:***, < 0.0001:****).

To further assess the five selected CARs in CD4 and CD8 T cells and to confirm their efficacy outside of the pooled assays, an extended in vitro repetitive stimulation assay was performed on each individually. 4-1BB, CD28, and a CD3ζ first-generation CAR were included as benchmarks due to their clinical relevance and characterization within the literature (6, 46). Proliferation was measured weekly using CTV over 33 days of repetitive antigen stimulation in two primary human donors (Fig. 4A to C, fig. S4A and B). Representative measurements for a single donor are shown alongside quantifications of the average change in mean fluorescence intensity (MFI) of the CTV stain (Fig. 4B and C). These arrayed stimulations resulted in relative proliferations similar to those observed in the pooled screen, with CD28 demonstrating less proliferation in CD4 T cells at later time points, CD40 generating strong proliferation in CD4 T cells, 4-1BB and BAFF-R demonstrating stronger late-stage proliferation through day 33, and KLRG1 displaying dramatically less cell division overall. Lastly, contrary to our pooled data, CD30 drove an initial burst of proliferation, primarily in CD4 T cells, but this was not sustained in later weeks. Overall, we found a high degree of correlation between our pooled and arrayed proliferation screens.

In addition to measuring proliferation by CTV dilution, we counted the number of T cells in culture every 3 days prior to restimulation with additional K562 tumor cells using flow cytometry and cell-counting beads. We used these counts to calculate the overall cumulative expansion of each CAR. Like the relative expansion measurements in our pooled screen, this considers both proliferation and resistance to cell death. In CD4 T cells from both donors, 4-1BB promoted a higher degree of cumulative expansion and persistence than CD28, in line with the clinical findings that 4-1BB CAR T cells are better long-term proliferators in vivo and are more resistant to exhaustion than CD28 CAR T cells (Fig. 4D) (4). However, we found that over 33 days, CD4 CAR T cells with CD40 costimulation doubled at an average of 1.8x the rate of those with 4-1BB or CD28 across both donors (Repeated Measures ANOVA, *p*=3.3×10^−3^), indicating, as in our pooled experiment, a heightened propensity for proliferation, resistance to cell death, or both during prolonged antigen stimulation.

A recent study found a strong association between enhanced mitochondrial metabolism and long-term proliferation (6). Therefore, we used single-cell energetic metabolism profiling (SCENITH), which uses oligomycin to inhibit mitochondrial oxidative phosphorylation (47) to determine its relative contribution to the overall metabolic output of each CAR T variant after 21 days in culture (fig. S4C). As expected, the CD3ζ-only CAR T cells demonstrated a low degree of mitochondrial metabolism, indicating an increased dependence on glycolysis. 4-1BB and CD28 CAR T cells were biased towards mitochondrial or glycolytic metabolism respectively, as previously noted in the literature (6). BAFF-R CAR T cells exhibited even higher mitochondrial dependence than 4-1BB after 21 days, in line with its long-term persistence in culture after repeated stimulations.

### T cells expressing BAFF-R and TACI CARs retain markers linked to persistence and demonstrate delayed exhaustion.

To determine the relationship between cell state and expansion of these CAR variants, we measured the expression of several exhaustion (programmed cell death protein 1 (PD1), lymphocyte activating 3 (LAG3), T-cell immunoglobulin mucin-3 (TIM3), CD39) and differentiation markers (CD62L, CD45RO, CD45RA, CD27, CCR7) throughout 33 days of repetitive stimulation (Fig. 4E, fig. S4D and E). BAFF-R CAR T cells exhibited slower upregulation of multiple exhaustion markers than 4-1BB and CD28, as shown by its overall lower number of markers on days 6 and 15 in both CD4 T cells and CD8 T cells (Fig. 4F, fig. S4D to F). Additionally, although the CAR T cells showed relatively similar differentiation over time, both BAFF-R and TACI CD8 CAR T cells sustained CD27 expression, in contrast to CD28 and 4-1BB, where CD27 expression progressively decreased (Fig. 4G, fig. S4G). We did not observe this trend in CD4 T cells (fig. S4G). CD27 has been linked to CD8 T cell survival after extensive proliferation, and resistance to terminal effector differentiation and contraction(48–51). Finally, as seen in our proliferation assays, expression of exhaustion and differentiation markers by KLRG1 CAR T cells were most similar to those in untransduced T cells, suggesting that a larger fraction of KLRG1 CAR T cells remain in a naive-like memory state (fig. S4D to H).

### Cytokine secretion and in vitro toxicity differ across signaling domains.

**Fig. 5.**
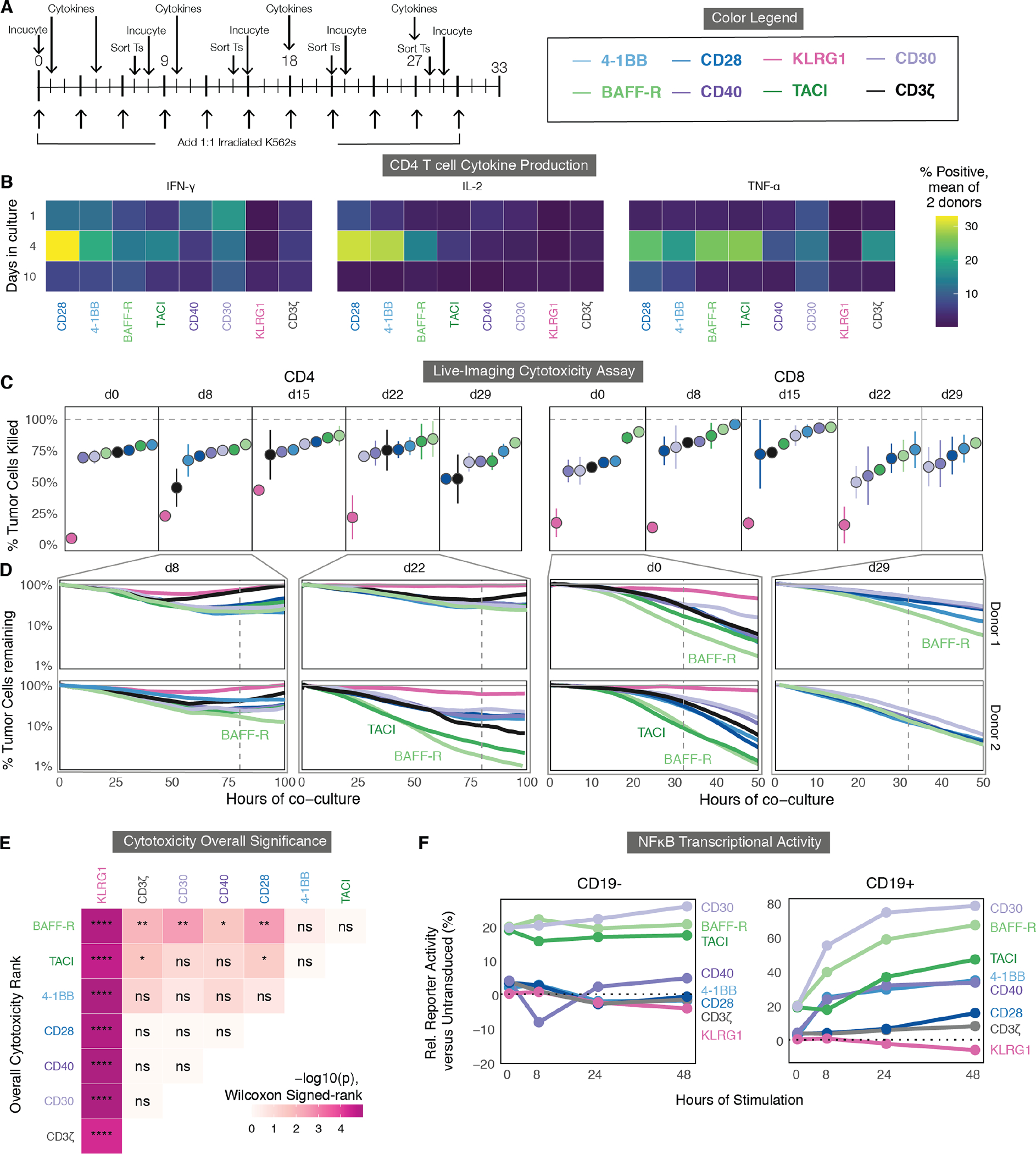
Comparison of signaling domains across measures of cytokine secretion, T cell signaling reporters, in vitro cytotoxicity, and in vivo solid tumor clearance. **(A)** Timeline for in vitro cytotoxicity and cytokine production assays. CD4 or CD8 T cells were transduced and stimulated as in Fig. 4A. Once weekly, a portion were stained for cytokine production as described in (B). For cytotoxicity assays, a portion of T cells (Ts) were sorted from the same co-culture by FACS, rested overnight, then cultured 1:1 with mKate+ CD19+ K562 cells and imaged every 60 minutes by Incucyte for the next 3 to 5 days (C and D). The color legend is shown on the right. **(B)** Mean cytokine production by CD4 T cells at 1, 4, and 10 days, measured across two donors by intracellular cytokine staining. Percentage of cytokine-positive cells was averaged between two donors. **(C)** Cytotoxicity of CD4 or CD8 CAR T cells sorted at the indicated days was quantified at 80 and 32 of co-culture respectively, by calculating the percentage of tumor cells at each time point relative to no T cells. CARs are ranked from least to most cytotoxic for each day. Error bars indicate the standard error calculated across donors. **(D)** Representative plots of cytotoxicity are shown for two donors’ CAR T cells sorted at day 8 and day 22 for CD4 T cells and day 0 and day 29 for CD8 T cells, plotting the percentage of mKate+ tumor cells remaining relative to a well with no T cells (gray). Vertical dashed lines indicate the time points analyzed in (C). **(E)** Overall significance is shown for the cytotoxicity of CD4 and CD8 T cells from two donors for all days indicated above. Data were analyzed using a Wilcoxon signed-rank test and FDR-corrected; p<0.05:*, p<0.01:**, p<0.001:***, p<0.0001:****; ns, not significant. **(F)** NFκB transcriptional activity was determined using a reporter Jurkat cell line transduced with each CAR and stimulated with either CD19- or CD19+ K562s. Samples were assessed by flow cytometry. The y-axis is relative (Rel.) to untransduced cells.

In addition to proliferation and persistence, we sought to measure differences in CAR T cell anti-tumor activity by cytokine secretion and cytotoxicity. We measured cytokine production in CD4 T cells in two human donors using intracellular flow cytometry after 1, 2, 3, 6 and 9 repeated stimulations in culture (Fig. 5A and B, fig. S5A). Most CAR T cells exhibited maximal cytokine production on day 4. Although comparisons can be made at early time points, none of the CAR T cells, including those containing the CD28 and 4-1BB costimulatory domains, produced substantial amounts of IFN-γ, IL-2, or TNF-α, as measured by intracellular staining after 3 or more in vitro stimulations (fig. S5A).

We next sought to assess the persistence of each CAR T cell’s cytotoxic capability in culture after intervals of repetitive antigen stimulation. To directly measure cytotoxicity in vitro, we used Incucyte live-cell imaging, which allows for long-term imaging of fluorescently labeled cancer and T cell co-cultures inside of an incubator. At multiple timepoints after the repeated antigen stimulations, we sorted either CD4 or CD8 T cells from the co-culture using fluorescence activated cell sorting (FACS) and let them rest overnight (Fig. 5A). The next day, we combined the sorted T cells with red-fluorescent K562 cancer cells and performed time-lapse live-cell microscopy to observe cell killing (Fig. 5C and D). We then quantified the percentage of cancer cells killed at 80 hours (CD4 T cells) or 32 hours (CD8 T cells) after each stimulation (Fig. 5C).

Although we observed differences in the cytotoxic capacity between the two donors, BAFF-R- and TACI-expressing showed superior cytotoxicity relative to other CAR T cells (Fig. 5C and D, fig. S5B). This was especially pronounced after multiple rounds of antigen stimulation within CD4 T cells. This enhanced cytotoxicity was statistically significant within both donors in CD4 T cells and CD8 T cells when comparing BAFF-R activity to CD28, CD40, CD30, KLRG1 and CD3ζ-only CAR T cells, and when comparing TACI to CD28, KLRG1, and CD3ζ-only CAR T cells (FDR < 0.05 by Wilcoxon Signed-rank test, Fig. 5E). We repeated these assays with two additional donors in CD4 T cells, comparing the top four domains (CD28, 4-1BB, BAFF-R, TACI), which further confirmed significantly enhanced CD4 cytotoxicity for BAFF-R versus the other 3 domains (FDR < 0.05, Wilcoxon Signed-rank test) (fig. S5C to E).

Additionally, we saw that KLRG1 had drastically reduced cytotoxicity, often only killing between 0 and 20% of K562 tumor cells, compared to the CD3ζ-only CAR, which killed approximately 75% of tumor cells at each timepoint (Fig. 5C to E). This was significant compared to the cytotoxic capabilities of all other CAR T cells (FDR < 0.0001, Fig. 5E). Combined with the proliferation, exhaustion, and differentiation data, this supports the hypothesis that KLRG1 dampens the CD3ζ domain’s function within a CAR T cell.

### Transcriptional reporters indicate differences in early signaling dynamics among the signaling domains.

Although most of our analyses indicated distinctions in CAR T cell phenotypes after prolonged periods of stimulation in vitro, we sought to determine if there were early differences in signaling upon the initial activation of each CAR that could help to understand the mechanisms behind these phenotypic differences. We transduced each of the CARs into three reporter Jurkat T cell systems that individually measured the transcriptional activity of activator protein 1 (AP-1), nuclear factor of activated T cells (NFAT), and nuclear factor kappa B (NFκB)(52). We then co-cultured these purified CAR-positive Jurkat reporters with CD19+ or CD19- K562 cancer cells for 8, 24, or 48 hours and measured their activity using flow cytometry (Fig. 5F, fig. S5F and G). We observed differences in basal and antigen-responsive transcription factor (TF) activity across the CARs, particularly in NFκB activity. Cells expressing BAFF-R, TACI, and CD30 CARs showed both accelerated dynamics and a higher total percentage of cells with NFκB activity (Fig. 5F), a TF that is closely associated with TNF receptor signaling (53, 54). These three CARs also upregulate AP-1 activity within the first 8 hours, two-fold more rapidly than any other costimulatory domain, whereas CD28 and 4-1BB CAR T cells expressed higher AP-1 without any antigen-based stimulation (fig. S5F). In addition to increased AP-1 signaling, NFAT reporter induction was more rapid and sustained in BAFF-R and TACI (fig. S5G). As expected, KLRG1 CAR T cells had reduced activity for all three TF reporters as compared to CD3ζ-only CAR T and untransduced T cells. Lastly, we also saw reduced basal AP-1 activity from CD40, which correlates with its lack of tonic signaling (fig. S5F). We did not include CD30 in any further analyses due to its high degree of tonic signaling and exhaustion marker expression in the arrayed in vitro experiments.

### Single-cell RNA-seq and CITE-seq characterize functional differences between CAR costimulatory domains.

**Fig. 6.**
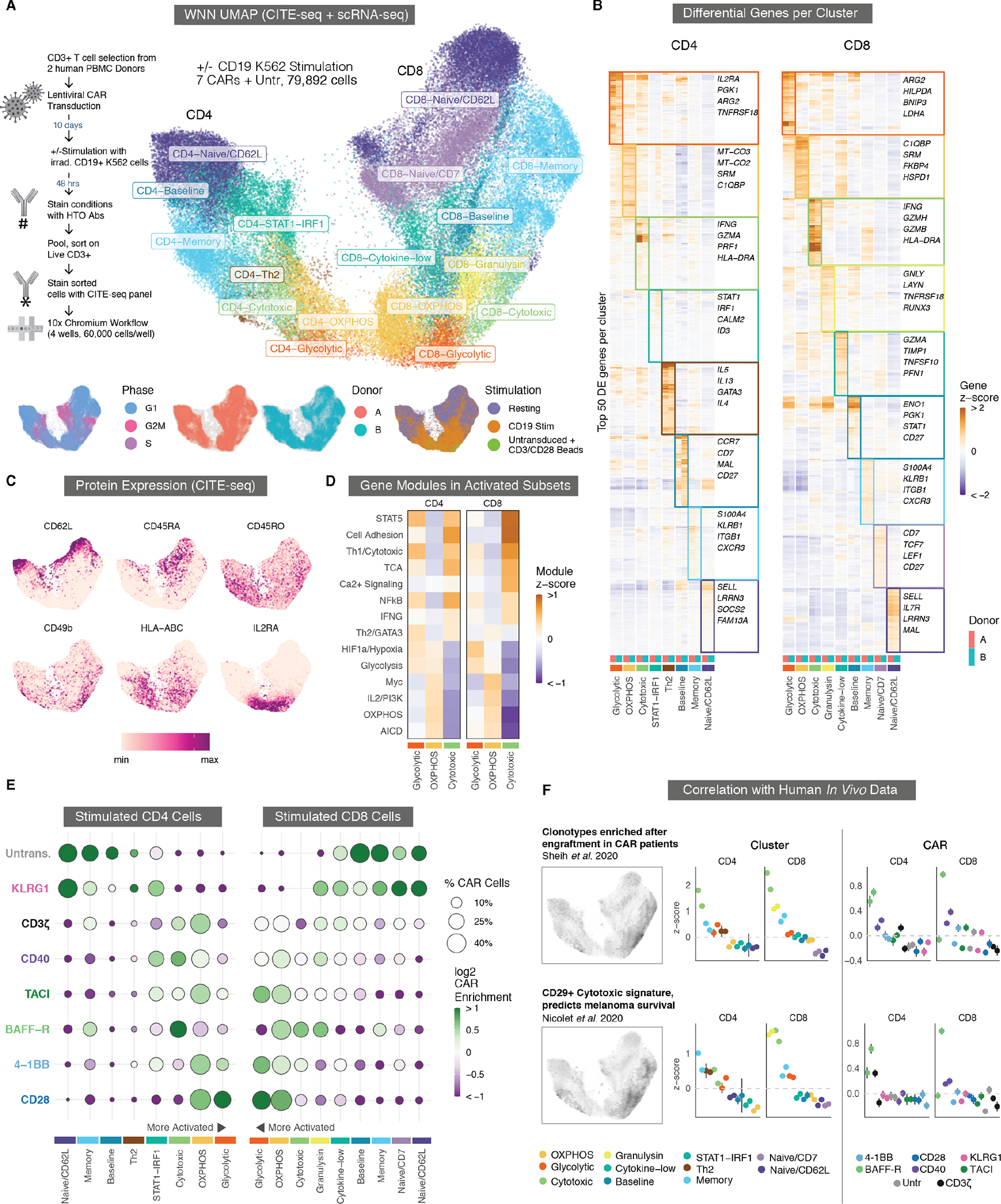
Single-cell RNA-seq and CITE-seq characterize functional differences between CAR costimulatory domains. **(A)** Weighted-nearest neighbor (WNN) Uniform Manifold Approximation and Projection (UMAP) embedding of scRNA-seq and CITE-seq data from stimulated and resting CAR T cells are shown for samples from two donors. UMAP separates into CD4 and CD8 lobes (left and right sides). Cells are colored by eight CD4 and nine CD8 phenotypic clusters. Bottom inset: Cells colored by cell cycle phase, donor identity, and stimulated versus resting cells. fig. S6A shows the UMAP faceted for each CAR and stimulation condition. **(B)** Heatmap of differentially expressed (DE) genes. For each cluster, the top 50 DE genes are ordered by hierarchical clustering of the pseudo-bulk expression z-scores for all clusters and donors. Genes in multiple clusters are only included for the cluster with the highest score. For each, four genes that are representative of the overall phenotype of the cluster are highlighted. **(C)** UMAP plots show relative CITE-seq expression for the surface expression of six markers associated with T cell differentiation and activation. HLA, human leukocyte antigen; IL2RA, IL-2 receptor subunit α. **(D)** Mean z-scores are shown for MSigDB gene modules associated with various aspects of T cell activation, metabolism, and signaling among the three major activated phenotypic clusters in CD4 and CD8 T cells. TCA, tricarboxylic acid cycle; HIF1a, hypoxia inducible factor 1 subunit alpha; OXPHOS, oxidative phosphorylation; AICD, activation-induced cell death. **(E)** Enrichment of stimulated CAR T cells containing different signaling domains within each phenotypic cluster. The size of each dot corresponds to the percentage of stimulated CAR T cells in a cluster and with a costimulatory domain. The color of each dot corresponds to the log2 enrichment or depletion of that CAR relative to others. Clusters are arranged with the most activated at the center to correspond to the (A) UMAP. Similar plots for resting cells and a per-donor breakdown are in fig. S6C and E, respectively. **(F)** Correlation of T cell gene signatures indicative of enhanced CAR T engraftment (top) and melanoma survival (bottom) with phenotypic clusters in CD4 and CD8 CAR T cells (middle column) or with CARs containing different costimulatory domains (right column). Cluster and CAR colors match those in (E). The two dots per group correspond to two separate donors. Error bars indicate 99% confidence intervals for the z-scores.

The marked differences between the CARs in the transcriptional reporter assay suggested that a deep and unbiased look into early transcriptomic signatures could explain their long-term functional differences in cytotoxicity, proliferation, and exhaustion which we observed throughout our repetitive stimulation co-culture. Previous studies have used single-cell RNA sequencing (scRNA-seq) to compare CARs containing CD28 and 4-1BB domains, identifying differences in signaling, metabolism, and differentiation (55). To achieve a comprehensive understanding of the phenotypic landscape of CAR T cells incorporating these new signaling domains, we evaluated single cell RNA expression and employed a cellular indexing of transcriptomes and epitopes by Sequencing (CITE-seq) antibody panel of 75 proteins (56) in order to map the unbiased transcriptome measurements onto well-studied T cell surface markers. We separately transduced each CAR, except for CD30, into bulk CD3 T cells from two peripheral blood mononuclear cell (PBMC) donors and performed 10x Chromium 3’ v3 scRNA-seq after two days in culture, either with or without stimulation provided by irradiated CD19+ K562 cells. (Fig. 6A).

We used a weighted-nearest neighbor graph-based clustering approach to combine both the CITE-seq and scRNA-seq data across 79,892 cells, followed by uniform manifold approximation and projection (UMAP) dimensionality reduction(57). This combined protein and RNA embedding separated the cells into well-defined CD4 and CD8 lobes (left and right), with resting cells at the outer edges and stimulated CD4 and CD8 cells in the center bottom, in distinct but adjacent regions (Fig. 6A, fig. S6A), suggesting that CD4 and CD8 CAR T cells converge towards a more similar activated phenotype after CAR stimulation. Additionally, although the cells were grouped into 8 CD4 clusters and 9 CD8 clusters (Fig. 6A and B), we noticed a pronounced mirroring of transcriptional programs across 5 pairs of clusters between CD4 T cells and CD8 T cells (Naive/CD62L, Memory, Cytotoxic, OXPHOS, and Glycolytic), and thus describe these clusters with matching labels (fig. S6B). Resting CAR T cells were found almost entirely in naive-like (Naive/CD62L and Naive/CD7) and memory clusters, whereas stimulated cells fell into three main clusters with differing cytotoxic and metabolic transcriptional and surface protein signatures (discussed below), as well as a few other clusters with phenotypes in between naive-like and fully activated (Fig. 6A to C, fig. S6C).

### Activated CAR T cells fall into 3 distinct clusters shared between CD4 and CD8 T cells.

In both CD4 and CD8 T cells, activated CAR T cells segregated into 3 distinct clusters we named Glycolytic, OXPHOS, and Cytotoxic, which differ in the transcription of several important metabolic genes and signaling pathways (Fig. 6B to D, fig. S6D). Cells in the Glycolytic cluster differentially upregulated transcription of genes involved in aerobic glycolysis (*ARG2*, *PGK1*, *LDHA*), the hypoxia inducible factor 1 subunit alpha (HIF1a) pathway (*SLC2A3*, *ENO1*, *ALDOC*), mitochondrial autophagy (*BNIP3*), protein markers involved in costimulation and T cell activation (*IL2RA*, *CD69*), and inhibitory receptors (*PD1*, *CTLA4*) (Fig. 6B and D, fig. S6E). Cells in the OXPHOS cluster upregulated genes involved in oxidative phosphorylation and arginine metabolism (*SRM*, *C1QBP*, *ATP5MC3*, *MT-CO3*), as well as the Myc, MTOR, and PI3K pathways (Fig. 6D). The third activated cluster, which we named Cytotoxic, had a strong inflammatory transcription signature, including high *IFNG* expression and expression of multiple granzymes and cytotoxic molecules including *GZMB*, *GZMK*, *GZMH*, *NKG7*, and *PRF1*. The Cytotoxic cluster was also enriched for transcripts associated with NFκB signaling, including *BIRC3*. Both CD4 and CD8 Cytotoxic cells also abundantly expressed both RNA and protein for major histocompatibility complex (MHC) class II and CD74, an MHC class II chaperone, as well as RNA and protein for a variety of integrins and chemokine receptors including *ITGB2* (CD29), *ITGA2* (CD49b), *ITGA4* (CD49d), *CXCR3*, and *CCR5*. The Cytotoxic cluster was more like Glycolytic than OXPHOS but shows distinct and overall lower expression of various inhibitory and activation protein markers, including IL2RA, OX40, 4-1BB, PD1, and glucocorticoid-induced TNFR-related protein (GITR); the Cytotoxic cluster also exhibited high expression of memory markers, including CD95 and CD45RO (Fig. 6C).

Having identified a variety of activation states and transcriptional signatures across CAR clusters, we next sought to identify those which were preferentially promoted by specific costimulatory domains (Fig. 6E, fig. S6A,C, and E). Although all 5 costimulatory domains were present in the three most activated clusters, CAR T cells that contained the CD28 and 4-1BB costimulatory domain were enriched in the CD4 and CD8 Glycolytic and OXPHOS clusters, whereas the BAFF-R CAR T cells were particularly enriched in the Cytotoxic clusters. BAFF-R was also the most enriched CAR in the Memory cluster after stimulation, which indicated that a larger proportion of CAR T cells containing this domain remained in a less activated and less differentiated state after CAR stimulation. This divergence in BAFF-R versus CD28 and 4-1BB enrichment was observed in both donors (fig. S6E).

### The cytotoxic cluster matches signatures of improved clinical response and CAR engraftment.

Neither mouse xenografts nor in vitro assays can fully recapitulate human in vivo biology and predict clinical outcomes of adoptive cellular therapies, but the unbiased and high-dimensional data acquired from single cell sequencing offers an opportunity to identify transcriptional signatures that correlate with positive clinical outcomes in patients. We found that the Cytotoxic cluster closely matches gene signatures associated with tumor infiltrating lymphocyte (TIL) and CAR efficacy identified in two recent studies. A study of clonal kinetics in patients undergoing CAR T immunotherapy identified a gene signature enriched in CAR T clones that preferentially expanded (IRF, increased relative frequency) in patients 1 to 2 weeks after infusion(28). We found that this IRF gene signature showed a distinct and significant overlap with gene expression in the CD4 and CD8 Cytotoxic clusters (p = 1×10^−9^), and that the BAFF-R CAR was enriched for this CAR expansion signature (Fig. 6F). A second set of studies by Nicolet and colleagues identified a T cell transcriptional signature that resulted in enhanced IFN-γ secretion and increased survival in patients with melanoma (29, 58). Their studies associated this cytotoxic gene signature with the integrin CD29. In the Cytotoxic cluster, particularly in BAFF-R CAR T cells, we see an overlap with this CD29 gene signature (Fig. 6F). We additionally see within both the Cytotoxic cluster and the BAFF-R CAR T cells an upregulation in RNA and protein expression of both CD29 and CD49d, which together form the heterodimeric VLA-2 integrin complex that is associated with more potent effector memory T cell responses (fig. S6D and F) (59, 60).

In addition to the increased expression of MHC genes and integrins, the Cytotoxic and Memory clusters also transcribed several receptors more typical of NK cells, including *KLRB1*, which encodes CD161. CD161 is expressed by a subset of T cells with characteristic tissue homing(61) and increased cytotoxicity (62), and recent work has shown that CD8+CD161+ T cells define a potent effector memory subset with enhanced CAR T cell efficacy (63). To explore this further, we turned to a recent study which identified a continuous gene expression gradient of lymphocyte innateness from T cells to NK cells(64). This innate lymphocyte gene expression program largely overlapped with the transcripts enriched in our Cytotoxicity and Memory clusters, transcripts enriched in the activated BAFF-R CAR T cells, and the clinical CAR T cell engraftment and CD29 and IFN-γ transcriptional signatures we identified from recent literature (fig. S6G). The unexpected overlap among these disparate data suggested a linkage between innate-like gene expression and beneficial cytotoxic CAR phenotypes. Overall, we showed that the CAR signaling domains we identified using CAR Pooling promote altered T cell states, including ones associated with beneficial anti-tumor responses.

### BAFF-R CAR T cells demonstrate enhanced in vivo efficacy.

To validate our observations in vivo, we tested the CAR T cells in two established tumor models known for their persistence and resistance to treatment. The first was an established epithelioid mesothelioma solid tumor model (M28), which is known to produce durable tumors that require CAR T cell persistence rather than rapid initial proliferation(52). We exogenously expressed CD19 on M28 cells and sorted for cells with CD19 expression comparable to K562 cells utilized in screening experiments. We injected NOD-*scid* IL2Rgamma^null^ (NSG) mice with 4×10^6^ M28 cells and 7 days later treated them with 6×10^6^ anti-CD19 CAR T cells. The *TRAC* locus of the CAR T cells was knocked-out using CRISPR to reduce potential graft-versus-host disease (GVHD) due to the long-term timescale of these experiments. To compare the rejection dynamics and ensure CARs targeting the exogenous CD19 antigen were similar to the previously established CARs targeting the endogenous ALPPL2 antigen, we set up a side-by-side in vivo experiment utilizing either anti-ALPPL2 4-1BB or anti-CD19 4-1BB CAR treatments. We saw no differences in tumor growth or rejection dynamics between CAR T cell treatments targeting the two antigens (fig. S7A and B).

**Fig. 7.**
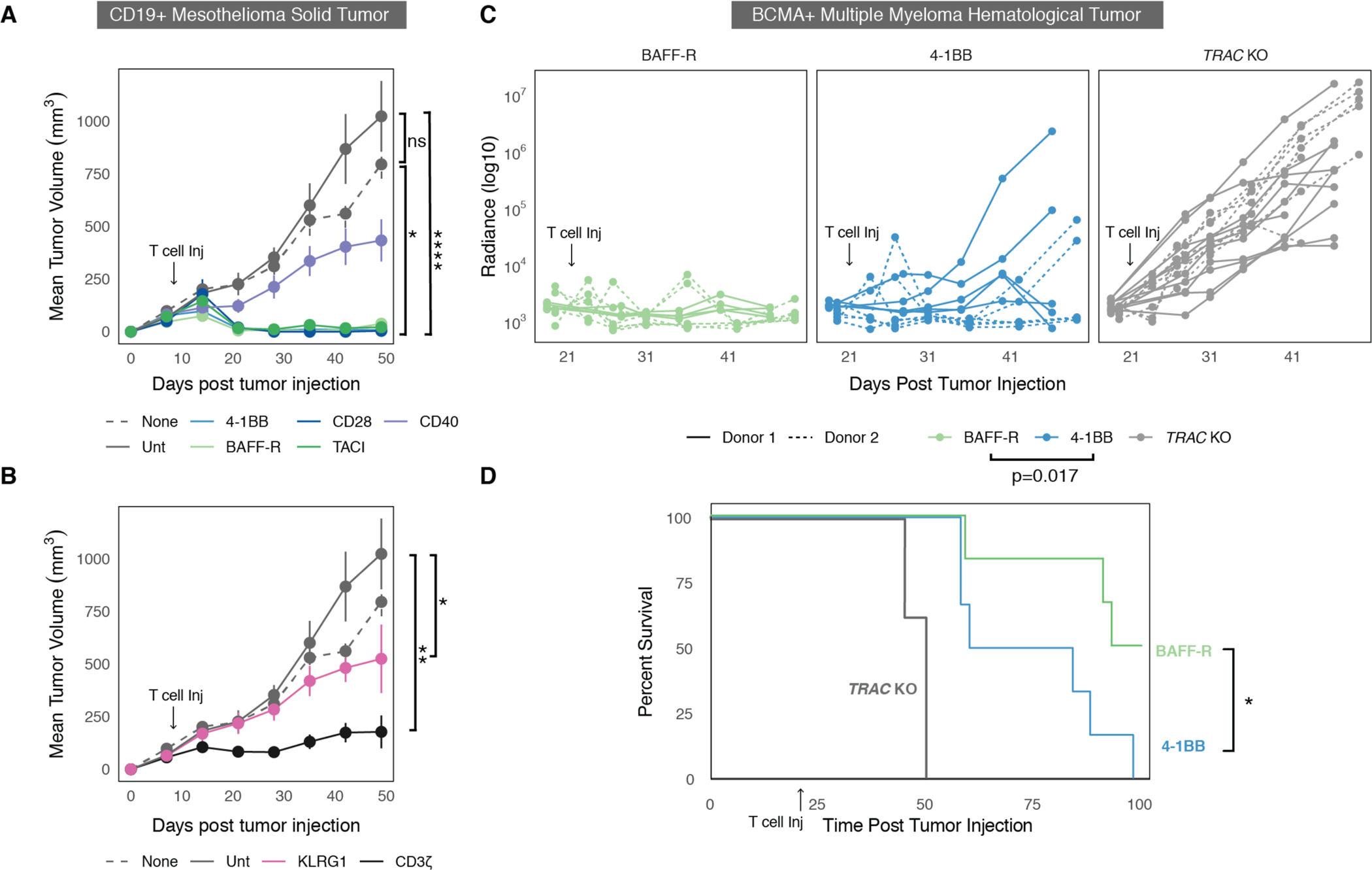
BAFF-R demonstrates potent anti-tumor in vivo activity in solid and hematological cancers. **(A)** Tumors were untreated, treated with untransduced T cells, or treated with engineered CAR T cells. Tumor size was monitored over 49 days post tumor injection. CAR T cells containing the potent costimulatory domains are shown, compared to untransduced T cells and no T cell controls. Data are representative of 5 in vivo experiments using 2 human donors. **(B)** Tumor size as in (A), showing a CD3ζ-only control and a KLRG1 inhibitory CAR, compared to untransduced T cells and no T cells. Error bars in (A and B) indicate standard error of the mean for tumor volumes across mice with the same treatment. **(C)** MM1S cancer radiance after luciferin injection was plotted over time for individual mice across CAR T cells generated from two donors. Tumors were measured by BLI every 7 days for a total of over 30 days. This was repeated independently in two donors. For panels (A to C), statistical analysis was done by t test on the normalized tumor volume area under the curve (AUC). **(D)** Survival curves are shown mice with MM1S cancer using T cells from a representative donor; mice were followed past 100 days (Mantel Cox test). For all panels, p<0.05:*, p<0.01:**, p<0.0001:****; ns, not significant.

Having confirmed similar tumor rejection between CAR T cells targeting the engineered and natural ligands, we compared the different CAR costimulatory domains head-to-head in the M28 CD19 model (Fig. 7A). We were unable to distinguish a difference between 4-1BB and CD28 CAR T cells at the time points shown here, despite the large amount of evidence from human studies that 4-1BB has increased long-term killing and persistence. Both 4-1BB and CD28, as well as the TACI and BAFF-R CAR treatments, exhibited similar tumor clearance and remission over 50 days. Additionally, CD40 demonstrated markedly diminished in vivo efficacy as compared to 4-1BB, CD28, TACI, and BAFF-R. This is likely related to CD40’s moderate in vitro cytokine production and cytotoxicity (Fig. 5B to E). Lastly, CAR T cells expressing KLRG1 mirrored the results from the in vitro cytotoxicity assay and showed markedly increased tumor burden compared to all other CAR T cells, including the CD3ζ-only CAR, while showing modest efficacy compared to untransduced T cells (Fig. 7B). The in vivo M28 data included are representative of 5 repeated experiments in two human donors (fig. S7C).

We also assessed CAR T activity in a multiple myeloma tumor model (MM1S) at a low “stress test” T cell dose. Multiple myeloma is a slow, incurable disease in which relapse is often a central clinical challenge. CAR T cells targeting BCMA are proving to be a transformative treatment against multiple myeloma (27). These primarily use 4-1BB costimulation, and although there have been substantial responses in patients treated with these CAR T cells, frequent relapse remains an issue (27). Thus, we engineered a clinical benchmark anti-BCMA 4-1BB CAR and replaced the costimulatory domain with the BAFF-R intracellular domain to determine if it could improve in vivo efficacy. We integrated the CAR into the *TRAC* locus using ssDNA non-viral integration (65) and injected 2×10^5^ CAR T cells intravenously into mice that received 0.5 to 1×10^6^ MM1S cells 21 days prior (0.5×10^6^ for Donor 1 and 1×10^6^ for Donor 2) (fig. S7D). We then measured tumors using bioluminescence for over 30 days. In data from two independent donors, the anti-BCMA BAFF-R CAR showed significantly improved control of MM1S cancer compared to 4-1BB (p = 0.017; t-test based on the normalized total cancer radiance AUC) (Fig. 7C). Anti-BCMA BAFF-R CAR T cell treatment significantly enhanced mouse survival over 100 days compared to the 4-1BB CAR 100 days post tumor cell injection (Mantel Cox test, p<0.05; Fig. 7D). Little variation was found in survival curves for a second human donor (fig. S7E). Swapping the costimulatory domain from 4-1BB to CD28 resulted in no enhancement of cancer control or survival (fig. S7E and F). Collectively, we show that BAFF-R-based CARs exhibit promise for clinical indications, as they show enhanced survival and tumor clearance in a mouse model of multiple myeloma and most closely phenocopy a transcriptional signature for enhanced T cell fitness and persistence that was independently identified within two human patient studies.

## DISCUSSION

Costimulation is essential for long-term T cell proliferation, differentiation, survival, and is known to enhance CAR T cell efficacy. A wide variety of known signaling domains beyond the canonical T cell costimulatory domains 4-1BB and CD28 have yet to be tested in CARs, and the extent to which signaling domains from other cell types can act upon T cells is poorly understood. Here, we identified several CAR signaling domains through pooled library screens in primary human T cells that enhance persistence or cytotoxicity over those used in the current generation of FDA-approved CARs. By screening 40 domains, we show the breadth of the signaling landscape within primary human CAR T cells. We observed that many of the most potent costimulatory domains measured belong to the TNF receptor family, which includes 4-1BB. 4-1BB signaling is known to increase T cell persistence and late stage proliferation relative to CD28(7), and our screen shows that this property extends to several other members of the TNF receptor family, especially CD40 and BAFF-R. Based on the results of our pooled screens, we selected CARs containing 4 new costimulatory domains from the TNF receptor family, as well as a candidate inhibitory CAR, and performed comprehensive characterization of their proliferation, persistence, differentiation, exhaustion, cytokine production, and cytotoxicity in vitro over several weeks of repetitive antigen stimulations. Characterizing these domains separately in CD4 T cells and CD8 T cells allowed us to identify effects specific to each cell type. We found that the BAFF-R and TACI signaling domains had heightened cytotoxicity, CD40 had heightened persistence in CD4 T cells, and KLRG1 can dampen ITAM-based signaling, preventing many hallmark features of T cell activation and differentiation.

To identify differences in gene expression elicited by our curated set of costimulatory domains, we explored the transcriptomic profile and surface protein expression driven by each CAR using scRNA-seq and CITE-seq both before and after antigen exposure. We observed differences in gene and protein expression between the CARs, based on their representation in clusters mapped to states of differentiation, activation, cytotoxicity, and metabolism, and also observed a distinctive mirroring of these clusters across the CD4-CD8 axis. Activated CAR T cells fell into three clusters which differed in their expression of key metabolic and cytotoxic genes. We named these activated clusters Glycolytic, OXPHOS, and Cytotoxic. The Cytotoxic cluster was enriched for CARs containing the BAFF-R costimulatory domain, which also showed strong anti-tumor and proliferative performance in our prior in vitro assays. We found overlap between this Cytotoxic cluster and gene signatures from several other recent studies of 4-1BB CAR infusion products and TIL efficacy in patients with cancer, suggesting that this signature may correlate with higher engraftment, persistence, and tumor rejection. Compared to 4-1BB, BAFF-R CAR T cells are approximately two-fold enriched (three-fold in CD4 T cells; 1.5-fold in CD8 T cells) for this gene signature, suggesting that a BAFF-R CAR infusion product would have a much larger fraction of cells in this hyper-effective state, potentially resulting in improved patient outcomes.

Finally, we compared CAR T performance in two in vivo models and found that our prior in vitro characterization, as well as our scRNA-seq and CITE-seq signature analysis, successfully identified CARs that promoted enhanced in vivo anti-tumor activity. In the xenograft model of epithelioid mesothelioma, BAFF-R- and TACI-based CAR T cells demonstrated equivalent tumor clearance to clinical benchmarks containing 4-1BB or CD28. In multiple myeloma, we observed enhanced tumor clearance and mouse survival upon anti-BCMA BAFF-R CAR T cell treatment with low stress-test doses, pointing to the potential for clinical development of BAFF-R-based CARs in multiple myeloma. Given high rates of multiple myeloma relapse due to limited CAR T cell persistence, an expanded set of CAR architectures with enhanced functions could improve clinical outcomes. (27).

Although we are primarily concerned here with identifying improved costimulatory domains, it is important to note that the KLRG1 CAR is not simply a negative control but a potential tool to control CAR activity, and could be useful for reducing cytokine release syndrome, abrogating on-target off-tumor activation, and even generating post-translational oscillations in CAR activity that would mimic natural T cell receptor signaling dynamics. Pooled screens can thus be employed not simply to find the optimal costimulatory domain hits, but rather as a discovery tool to understand how different receptor signals alter T cell biology and how these signals can be modulated to manipulate T cell function beyond optimizing a single chimeric antigen receptor.

Although CAR Pooling allowed us to compare large numbers of signaling domains directly and efficiently, our study has limitations. Discrepancies can arise between the performance of domains in a pooled versus an arrayed setting, as demonstrated by our observation that CD30 drives long-term expansion in the pooled screen but rapid exhaustion and tonic signaling in the arrayed screen. These differences could be due to paracrine effects by neighboring CAR T cells in the pooled screens. We also observed differences between in vitro and in vivo performance, such as the lack of in vivo anti-tumor efficacy for CD40. Although NSG mice do not encompass the complexity of the natural tumor microenvironment, they apply additional selection criteria that our in vitro cytotoxicity assays do not. To address such discrepancies, future CAR Pooling assays could also be performed directly in vivo and utilizing mouse models with fully reconstituted immune systems. Measurements associated with cytotoxicity (cytokine production, granzymes, or CD107a) could also be performed ex vivo after intratumoral expansion of the library. Lastly, our study primarily focuses on varying the costimulatory domains within a single version of the CAR architecture utilizing the CD19 scFv. Although our final in vivo data includes BCMA targeting, it is likely that different hits can arise from performing CAR Pooling with different scFvs, juxtamembrane domains, or ITAM-containing domains other than CD3ζ. Despite these limitations, CAR Pooling has resulted in the design of receptors such as the BAFF-R CAR and can be used to explore other critical domains that make up the CAR architecture.

Much of the existing literature tends to compare CARs one-dimensionally as more or less effective overall; here, we instead observed that individual CARs often excelled within specific assays or when expressed in different T cell types. This multidimensionality of costimulation was somewhat unexpected, reinforcing that what is usually referred to as a monolithic “signal 2” is instead a variety of heterogeneous pathways, sometimes in opposition, which have the potential to be individually tuned to optimize different aspects of T cell function. This also suggests that future engineering could isolate individual signaling motifs to enhance specific T cell phenotypes and create synthetic combinatorial domains which are optimized for specific scFvs, tumor types, or to better combat the functional deficiencies seen in CAR T cells for solid tumors. Finally, in addition to engineering better therapeutics, future high-throughput studies will help to elucidate the design rules for synthetic receptors and signaling motifs, generate powerful new tools to manipulate T cells for basic immunology research, and lead us to a greater understanding of T cell differentiation, development, and immune-related disease.

## Materials and Methods

### Study design

The aim of this study was to develop a new multiplexed system for comparing many genetic CAR architectures, and to identify and characterize new costimulatory domains for improving CAR efficacy during a repetitive chronic antigen challenge. We started by generating a library of second-generation CAR T cells using 40 different signaling domains. This library was measured using several parallel multiplexed screens in a repetitive in vitro stimulation model. For the pooled screens, two or three donors were used, and CD4 and CD8 T cells were transduced separately for each. Measurements were removed for an individual construct/replicate if fewer than 500 cells were sorted for that construct. Seven of these domains were chosen, and two separate donors were used to validate and further characterize them. All donor PBMCs were collected from healthy donors. For all in vivo experiments, mice were assigned to treatments randomly and the researchers were blinded until after data collection. A table of all reagents with source and catalog number are listed in table S2. Human T cell donor information including sample processing and demographic details for all experiments are included in Data file S1.

### Library construction

Costimulatory intracellular domains (ICDs) were synthesized by Integrated DNA Technologies, amplified, and individually cloned of the C-terminal portion of a CAR construct (CD3z-2A-GFP) and sequence-verified. Each domain was miniprepped separately and pooled at 1:1 molar ratio. A fragment containing an SFFV promoter and N-terminal portion of the CAR up until the costimulatory domain was inserted in front of the pooled ICD plasmid library using Golden Gate Assembly and electroporation. This was then digested and inserted by restriction cloning and electroporation into a pHR-SIN lentiviral backbone. We ensured at least 1000x coverage at each electroporation step.

### Lentivirus production and concentration

To produce lentivirus, Lenti-X 293T cells (Takara Bio) were transfected with a transgene expression vector and the viral packaging plasmids pCMVdR8.91 and pMD2.G using TransIT-Lenti Transfection Reagent (Mirus Bio LLC). K562 tumor cells were originally obtained from American Type Culture Collection (ATCC), CCL-243. M28 tumor cells were originally obtained from B. Gerwin’s laboratory at the National Cancer Institute. Both M28 and K562 cell lines were transduced to exogenously express CD19 using lentiviral transduction and flow cytometry sorting to obtain a pure population with CD19 expression within a one log maximum width. Lentiviral concentration was performed 72 hours after Lenti-X 293T cell transfection through collection and filtration of the viral supernatant. Per 20mL of viral supernatant we added 4.58mL 50% PEG 8000 (final concentration 8%) and 2mL of 4M NaCl (final concentration 0.3M) for 6 to 8 hours. We pelleted the virus by spinning down cells at 3500rpm for 20 minutes at 4°C, decanted, and resuspended the pellet in 200μL phosphate-buffered saline (PBS). We then snap froze aliquots on dry ice for storage in a −80°C freezer.

### In vitro T cell production

Blood samples in the form of leukopaks were obtained from healthy male and female volunteers through STEMCELL Technologies. For in vitro experiments, T cells were isolated from PBMCs using CD4 or CD8 negative selection kits and frozen. For in vivo experiments, T cells were isolated from PBMCs using CD3 negative selection kits and frozen. We stimulated the T cells 24 hours after thawing with 25μL of CD3/CD28 Dynabeads (Thermo Fisher Scientific) per 1×x10^6^ T cells (1:1 ratio). Concentrated lentivirus was added 48 hours after thawing to reach a transduction rate of under 15% for the pooled library experiments, between 30 to 50% for the arrayed screens, and between 65 to 90% for the in vivo experiments. Virus was removed within 18 hours of addition and cells were expanded. Five days after thawing, the CD3/CD28 Dynabeads were removed by magnetic separation and cells were sorted for GFP expression at least half a log higher than the negative population and spanning no more than a log in MFI. Cells were plated at 0.5×10^6^ cells/mL and split every three days to this density until 10 to 14 days after thawing. All references to T cell media indicates usage of X-VIVO 15 + 5% human AB serum + 10mM N-acetylcysteine (NAC) neutralized with 1N NaOH + 0.5% penicillin/streptomycin + 1X beta-mercaptoethanol.

### K562 cell irradiation

Live K562 cells (ATCC CCL-243) were grown up in T182 flasks until confluent. Cells were resuspended to 10×10^6^/mL on ice and irradiated using a Cesium-137 irradiator for 20 minutes (about 200 rad/min) in a 50mL falcon for a total dose of approximately 4,000 Rads. Cells were then aliquoted and frozen in Iscove Modified Dulbecco Media (IMDM) containing 10% dimethyl sulfoxide and 10% fetal bovine serum (FBS) in liquid nitrogen until needed.

### Repeat In Vitro Stimulations with K562 cells

For the pooled experiments, we started stimulation with tumor cells on day 10; for the arrayed experiments we started stimulation with tumor cells on day 14. To stimulate the T cells, we combined them 1:1 with irradiated K562 cells that either expressed or did not express surface human CD19 and plated at a density of 5×10^5^ K562 cells/mL and 5×10^5^ T cells/mL. Every three days, the cultures were centrifuged, resuspended, and counted using flow cytometry to split the T cells and add more irradiated K562 cells. We counted the T cells by adding an aliquot of the resuspended culture to CountBright beads and calculating the number of T cells through analysis on the BD X-50 Flow Cytometer and using FlowJo software. The cultures were restimulated with the addition of a new bolus of irradiated K562 cells at a 1:1 ratio to total T cells in each culture. They were replated at the density of 5×10^5^ T cells/mL. This was repeated for a total of 3 to 33 days.

### CTV dye labeling and analysis

CAR T cells were isolated from cultures, resuspended, and washed with PBS. The cells were resuspended to 1×10^6^ cells/mL in a 5μM solution of CTV in PBS and incubated at room temperature for twenty minutes in the dark. We then added 5mL of T cell media on top for every 1×10^6^ cells that were stained and incubated another 10 minutes in the dark. Then, cells were pelleted by centrifugation at 500g for 5 minutes, resuspended, and plated in T cell media at 1×10^6^ cells/mL. To stimulate proliferation, we plated an equal amount of K562 cells relative to the total number of T cells in each well, with or without CD19 expression, as described above. We assessed the proliferation 3 days after stimulation for CD4 T cells by flow cytometry. For CD8 T cells, we restimulated T cells with an additional dose of irradiated K562 cells day 3 after the initial stimulation and stained for flow cytometry on day 4 after the initial stimulation. To determine proliferation after prolonged co-culture, rather than restaining, we kept a proportion of the cells separate in culture and stained on day 9 (CTV2), day 18 (CTV3), or day 27 (CTV4) post-initial stimulation. Therefore, all CTV-stained populations were stained only once with CTV dye. We assayed proliferation on day 16 (CTV2), day 24 (CTV3), or day 33 (CTV4) by flow cytometry using a FACSymphony X-50 cytometer and FlowJo for analysis.

### CD69 staining and analysis

To determine the degree of activation induced by each CAR, we stimulated antigen-naive T cells with irradiated K562 cells, either with or without CD19 expression, in a 1:1 ratio. After 24 hours, the cells were centrifuged at 500g for 5 minutes, washed twice in flow buffer (PBS + 2% FBS), and stained with anti-CD69 brilliant ultraviolet (BUV) 395 antibodies diluted 1:200 in flow buffer at 4°C for 20 minutes. The cells were washed twice with flow buffer and analyzed using a FACSymphony X-50 cytometer and FlowJo for analysis.

### Analysis of cytokine production by CAR T cells

To determine the degree of cytokine production upon activation of each CAR, we stimulated antigen-naive T cells with irradiated K562 cells, either with or without CD19 expression, in a 1:1 ratio. Three days after, we replated the co-culture with a secondary bolus of K562 cells as described above. 12 hours later, we added 2x Brefeldin A and incubated the cells for an additional 6 hours. We centrifuged the cells at 500g for 5 minutes, washed twice in flow buffer, and stained with anti-CD4 phycoerythrin (PE) 1:200 or anti-CD8 PE 1:200 antibodies at 4°C for 20 minutes. We washed the cells twice with flow buffer and added 100μL of fixative (50μL of flow buffer + 50 μL of Invitrogen IC fix) to each well. We incubated at room temperature for 1 hour in the dark. After fixation, we spun the cells at 600g for 5 minutes and resuspended in Cytolast for continued staining the next day. To permeabilize the cells we added 200μL of 1x permeabilization buffer to each well, immediately spun at 600g for 5 minutes, and stained for intracellular antigens with anti-IL-2, anti-TNF-ɑ, and anti-IFN-γ antibodies diluted in permeabilization buffer. Cells were stained in a total volume of 50μL at room temperature for 30 minutes in the dark. Cells were washed twice with permeabilization buffer, resuspended in flow buffer, and analyzed using a FACSymphony X-50 cytometer and FlowJo for analysis.

### Incucyte analysis

50 μL 5 μg/mL of fibronectin was dispensed to each utilized well of a 96-well plate. The plate was incubated for 60 minutes at room temperature, and fibronectin was removed, followed by another 60 minute incubation at room temperature. Both CAR T cells and live K562 target cells (either expressing mKate and CD19 or only mKate) were spun down and resuspend in Jurkat media + 30 U/mL IL-2; Jurkat media (RPMI-1640 + 10% FBS + 1% Penicillin/Streptomycin + 1X Glutamax) has less fluorescence than media based on X-VIVO-15. Cells were counted and diluted to 2.5×10^5^/mL each, and 100 μL of each (T cell and targets) was added to each well for a final assay volume of 200 μL. Each condition was done in duplicate so long as sufficient cells were available. We allowed plates to settle at room temperature for 30 minutes before beginning the Incucyte assay. Images were taken every 60 minutes using the Incucyte software over the course of the experiments. See relevant figures for total assay times, which varied between conditions.

### SCENITH Staining and Analysis

After T cell preparation for in vitro stimulation assays (see above), we pulled cells out at the designated timepoints during the repetitive stimulation assay to assess cell metabolism by flow cytometry. We stimulated 50,000 T cells with 50,000 K562 cells in 100μL within a 96 well U bottom plate and incubated them for 24 hours. We then added inhibitors for 30 minutes at 37°C followed by puromycin for 15 minutes at 37°C as outlined in Argüello *et al.* (47). To assess cell viability, we washed cells with cold PBS and stained with Zombie near infrared (NIR) dye at a 1:1000 dilution for 30 minutes at room temperature. We washed cells once in flow buffer and stained in 50μL of surface stains composed of CD4 BUV395 (1:200) and CD8 Pacific Blue (1:200) for 20 minutes on ice. We washed cells in 100μL fixation solution from eBioscience IC Stain Kit. We then fixed cells for 30 minutes at room temperature, washed two times in permeabilization buffer, and then stained with a 1:100 dilution of anti-puromycin alexa fluor (AF) 647 in permeabilization buffer for 30 minutes on ice. Cells were washed twice in flow buffer and resuspended in flow buffer for analysis using FACSymphony flow cytometer. Analysis was completed on FlowJo as described in Arguello *et al.* (47).

### DNA extraction and sequencing

After fluorescence activated cell sorting assays, after in vitro growth with target cells, or after transduction as a library abundance baseline measure, T cells containing the CAR library were centrifuged, supernatants were removed, and pellets were frozen at −80°C. Subsequently, genomic DNA was prepared from cells using either the Machery-Nagel Nucleospin Tissue XS column, Machery-Nagel Nucleospin column, or the Nucleospin 96 Tissue extraction plate. Manufacturer protocols were followed except for the addition of 10 μg polyadenylated RNA to each sample to increase yield.

After gDNA prep, Picogreen and a plate-based fluorescence reader were used to quantify the extracted genomic DNA. Initial polymerase chain reaction (PCR) amplification of the costimulatory domain region from the different samples (PCR1) were done in 3 batches with differing numbers of cycles (12, 16, or 22 cycles) depending on the genomic DNA concentration. PCRs were performed with Takara ExTAQ to allow for maximum template concentration to be used in the PCR reaction. Reactions were done in 70 μL with between 200 and 1000 ng of DNA used as template depending on the batch as described above.

For the subsequent PCR to add Illumina barcodes and adapters to the products (PCR2), all products from PCR1 were diluted 15x and 25 μL of template was used in a 50 μL reaction with Takara ExTAQ. Different forward and reverse primers were used for each sample for PCR2 to add unique custom Illumina I5 and I7 barcode sequences to each sample. Finally, PCR2 products were again quantified using Picogreen in a plate-based fluorescence reader. These products were pooled at 1:1 molar ratio, diluted, loaded, and run on a MiniSeq 2×150 cycle cartridge using the standard manufacturer protocols. After demultiplexing, CAR costimulatory domain sequences in FASTQ format for each sample were adapter-trimmed, sorted, deduplicated, and aligned using custom python scripts and BWA-mem. These alignments were then converted into count tables and analyzed using DESeq2 and custom R scripts (DOI:10.5281/zenodo.7062819).

### Calculations for in vitro cumulative expansion over repeat stimulations

Each culture of CAR T cells was produced as described above in “In vitro T cell production” methods section. T cells were then stimulated 1:1 with irradiated K562 cells as described and counted every three days using CountBright beads at a ratio of 1:20 in volume of beads to cells. This mixture was then run on an X-50 Fortessa and analyzed using BD FACSDiva for the number of CAR positive T cells in culture (analyzed using the T2A-GFP).

### T Cell purification and transduction for scRNA-seq

Experiments for each donor were performed separately on different days. CD3 T cells were purified from PBMCs extracted from either leukopaks or Trima residuals, as described above. CARs were then lentivirally transduced into bulk CD3 T cells, using the same methods as previously described, and five days after transduction, T cells were FACS-sorted using on GFP marker expression based on a range of a one log away from the mean expression across all constructs. Untransduced T cells were not sorted.

### T cell stimulation and 10x preparation for scRNA-seq

Five days after sorting (total of 10 days after transduction), 2×10^6^ cells were plated with either 2×10^6^ irradiated CD19+ K562 cells (as described above) or replated in fresh media without K562 cells, at a density of 1×10^6^ T cells/mL, for a total of 18 conditions per T cell donor: 6 second-generation CARs (excluding CD30), a CD3ζ-only CAR, and untransduced T cells, both with and without K562 co-culture. For the second donor, a CD3/CD28 Dynabead-stimulation condition was also performed with untransduced cells, following instructions from the manufacturer. A K562-only control sample was also generated for both donors. After co-culture for 48 hours, cells in each condition were individually counted and stained CD3, DRAQ7, and with unique combinations of two TotalSeq-B hashtag-oligo (HTO) antibodies (BioLegend) following the manufacturer’s instructions (table S2). After staining, the cells were pooled at approximately equal ratios based on counts taken prior to staining. The pooled cells were then sorted based on forward scatter (FSC) and side scatter (SSC), CD3, GFP, and DRAQ-7-negative gates to remove dead cells and irradiated K562 cells. Finally, the sorted cells were stained using the BD Bioscience ABseq antibody panel per the manufacturer’s instructions and loaded and processed using the standard Chromium V3 3’ sequencing pipeline. Two lanes were loaded per donor at 60,000 cells per lane. Cells were able to be loaded at this high density because doublets could be identified based on HTO barcode collisions between samples. One 200-cycle Novaseq S4 lane of Illumina sequencing was performed per donor (approximately 6e9 reads in total).

### scRNA-seq data loading and cleaning

Unique molecular identifier (UMI) counts were generated using CellRanger v5.0.1 using the standard settings and imported into Seurat v4, R v4.0.4, Rstudio v1.4. Barcode combinations were deconvolved using custom scripts and potential doublets were identified and removed based on barcode collisions (data at DOI:10.5281/zenodo.7063644, code at DOI:10.5281/zenodo.7062819.). CD4 and CD8 T cells were identified based on RNA and antibody-derived tags (ADT) expression of CD4 or CD8, and double-positive cells were removed from consideration. Cells whose UMIs came from mitochondrial genes greater than 25% of the time were removed. A total of 79,892 cells remained after removing potential doublets and cells enriched for mitochondrial RNA.

### Multidonor and multimodal scRNA-seq data integration and clustering

Generation of UMAP embedding on both scRNA-seq and CITE-seq data was performed using Seurat v4’s SCTransform and FindMultiModalNeighbors functions. Regression was done on cell cycle genes (Seurat’s *cc.genes*). Then, FindClusters was used to generate initial clusters with algorithm 3, cluster resolution 1.3. For initial data exploration purposes, this UMAP embedding and clustering procedure was performed separately for activated CD4 T cells, activated CD8 T cells, all CD4 T cells, all CD8 T cells, and finally with all cells (the latter generating the embedding shown in Fig. 6A). Initial clusters were identified, then categorized and consolidated into the final clusters in Fig. 6A, based on curation of the most differentially expressed genes and proteins, an extensive search of the literature, using Vision, Gene Set Enrichment Analysis (GSEA) and the Molecular Signatures Database (mSigDB), and by hierarchical clustering of average gene expression across clusters.

### M28 in vivo experiments:

All in vivo tumor experiments used sex and age matched NOD.Cg-Prkdc^scid^Il2rg^tm1Wjl^/SzJ (NSG) (RRID:IMSR_JAX:005557) mice between 8 and 12 weeks of age. Researchers were blinded to both previous tumor measurements and treatment paradigm, and all work was conducted under a protocol approved by the UCSF Institutional Animal Care and Use Committee (AN194345-01C). Four days prior to tumor injection, M28 CD19+ tumor cells were split and 0.75×10^6^ cells were plated in one T182 flask for every 2.5 mice for propagation. On day 0, M28 CD19+ cells were trypsinized, counted, and resuspended in RPMI-1640 at 40×10^6^ cells/mL. We injected NOD-*scid* IL2Rgamma^null^ (NSG) mice with 4×10^6^ M28 cells subcutaneously on the right flank and measured the initial tumor growth by caliper 6 days later. Groups were split up so that they have even tumor volume variance. A dose of 6×10^6^ GFP positive anti-CD19 CAR T cells (generated either with or without TRAC-knockout, see below) were injected on the 7^th^ day post tumor injection intravenously by tail vein. Tumors were measured by caliper every 3 to 7 days for a total of 30 to 50 days.

To generate T cells for the ALPPL2 experiment, we produced T cells as described above using 30μL concentrated virus per 1×10^6^ T cells. This achieved a transduction rate between 65 and 90%, as assessed by flow cytometry. T cells were injected as described above 10 days after initial thaw.

The following describes the T cell production utilized when knocking out the *TRAC* locus for T cell receptor downregulation and prolonged in vivo timelines. We stimulated bulk CD3+ T cells isolated from human PBMCs 4 hours after thawing with 25μL of CD3/CD28 Dynabeads (Thermo Fisher Scientific) per 1×10^6^ T cells (1:1 ratio). 24 hours after stimulation, concentrated lentivirus was added to the T cells at 30μL per 1×10^6^ T cells. 48 hours after stimulation, the lentivirus was washed off and Dynabeads were removed by magnetic separation. Cells were replated at 1×10^6^ cells/mL. After an additional 24 hours, we electroporated Cas9 and guide ribonucleoprotein (RNP) to knock out the *TRAC* locus with approximately 98 to 99% efficiency as assessed by anti-CD3 BV711 staining (1:200) using a FACSymphony X-50 flow cytometer and analysis using FlowJo. Cells were plated at 1×10^6^ cells/mL and split every three days to this density until 10 days after thawing. They were then injected as described above.

### MM1S in vivo experiments:

All MM1S experiments used sex and age matched NOD.Cg-Prkdc^scid^Il2rg^tm1Wjl^/SzJ (NSG) (RRID:IMSR_JAX:005557) mice between 8 and 12 weeks of age. Researchers were blinded to both previous tumor measurements and treatment paradigm, and all work was conducted under a protocol approved by the UCSF Institutional Animal Care and Use Committee (AN194345-01C). Mice were inoculated with 0.5×10^6^-1×10^6^ MM1S-luciferase^+^ cells by tail vein injection (0.5×10^6^ for donor 1, 1×10^6^ for donor 2). Three weeks after, mice were randomized post bioluminescence imaging (BLI) for tumor radiance measurements. 200,000 BCMA-targeting CAR T cells were intravenously injected into the tail vein. Bioluminescence was measured with the Xenogen IVIS Imaging System (Xenogen) about 10 to 12 minutes after injecting 200μL of luciferin intraperitoneally and analyzed utilizing the Living Image software.

For the *TRAC* knockin CAR T cell generation for MM1S tumor experiments, long single-stranded DNA was manufactured at large scale using PCR with a biotinylated primer followed by magnetic bead purification, as described previously(65). Bulk T cells were isolated from PBMCs as described above and stimulated 4 hours later 1:1 with CD3/CD28 Dynabeads (Thermo Fisher Scientific). 48 hours after stimulation, Dynabeads were removed by magnetic separation and cells were electroporated with Cas9 and guide RNP to knock out the *TRAC* locus with approximately 98 to 99% efficiency as described above. Long ssDNAs were also added to the electroporation mixture to integrate the CAR into the *TRAC* locus. Cells were plated at 1×10^6^ cells/mL and split every three days to this density until 10 days after thawing. 200,000 cells were then injected intravenously by the tail vein 21 days after MM1S injection.

### Statistical Analysis

Raw, individual level data for experiments where n<20 are presented in data file S2. All relevant code used for statistical modeling, summarization, and visualization is available in our GitHub repository, (DOI:10.5281/zenodo.7062819). In all cases, multiple testing corrections were performed using the Benjamini–Hochberg procedure. For Fig. 2C, 3C, and 4D, repeated-measures mixed effects linear models were constructed in R using the *nlme* package to assess statistical significance for relative expansion over time across the CAR signaling domains across different donors, time-points, and between CD4 and CD8 subsets, where applicable. Model selection was performed using Akaike information criterion (AIC)/Bayesian information criterion (BIC) and an autoregressive 1 (AR1) correlation structure was chosen. For Fig. 2C, CD4 and CD8 subsets were considered separately, whereas for Fig. 3C, they were combined. Each metric along the y-axis of Fig. 3A was converted into a set of z-scores within each donor, replicate, and T cell subset; metrics were checked for adherence to normality using the Anderson-Darling method and visually using Q-Q plots. Z-scores were then combined across donors and replicates using Stouffer’s method, then a one-sample *z*-test was performed on each domain. Scores were false discovery rate (FDR)-corrected using the Benjamini-Hochberg procedure. A similar model was fit to the CD40/CD28/4-1BB arrayed expansion comparison in Fig. 4D. For Fig. 1E, 4F, 4G, and fig. S4F, repeated-measures analysis of variance (ANOVA) models were constructed in R using the *aov* function to assess statistical significance. Donor and CD4 or CD8 T cell subsets, where applicable, were used as error strata such that aov(measure ~ car * day + Error(donor), where car is the costimulatory domain identity, day is the measurement timepoint, and donor is the name of the donor.

## Supplementary Material

Data File S2

Data File S1

1

## Data Availability

All data associated with this study are in the paper or supplementary materials, including the raw data for experiments where n<20 in Data file S2. scRNA FASTQ files are available on the SRA, BioProject PRJNA878854. Additional raw data, including FCS files, DNA sequences for plasmids and constructs, incucyte measurements, barcode counts, and Rdata object files are located at 10.5281/zenodo.7063644. Processed data and code used for analysis and figure generation is available at 10.5281/zenodo.7062819. All generated research materials are available on request by contacting the corresponding author.
