# Supplementary material for: Pooled screening of CAR T cells identifies diverse immune signaling domains for next-generation immunotherapies
Source: Sci Transl Med. Author manuscript; Available in PMC 2023 Feb 19. (PMC9939256; doi:10.1126/scitranslmed.abm1463)
Supplement: 1 [file NIHMS1869253-supplement-1.pdf]

Supplementary Materials for

Pooled screening of CAR T cells identifies diverse immune signaling domains for next-generation immunotherapies

Daniel B. Goodman†, Camillia S. Azimi†, Kendall Kearns, Alexis Talbot, Kiavash Garakani, Julie Garcia, Nisarg Patel, Byungjin Hwang, David Lee, Emily Park, Vivasvan S. Vykunta, Brian R. Shy, Chun Jimmie Ye, Justin Eyquem, Alexander Marson, Jeffrey A. Bluestone, Kole T. Roybal*

Correspondence to: kole.roybal@ucsf.edu

This PDF file includes:

Figs. S1 to S7
Table S1 and S2
Legend for data file S1 and S2

Figure S1

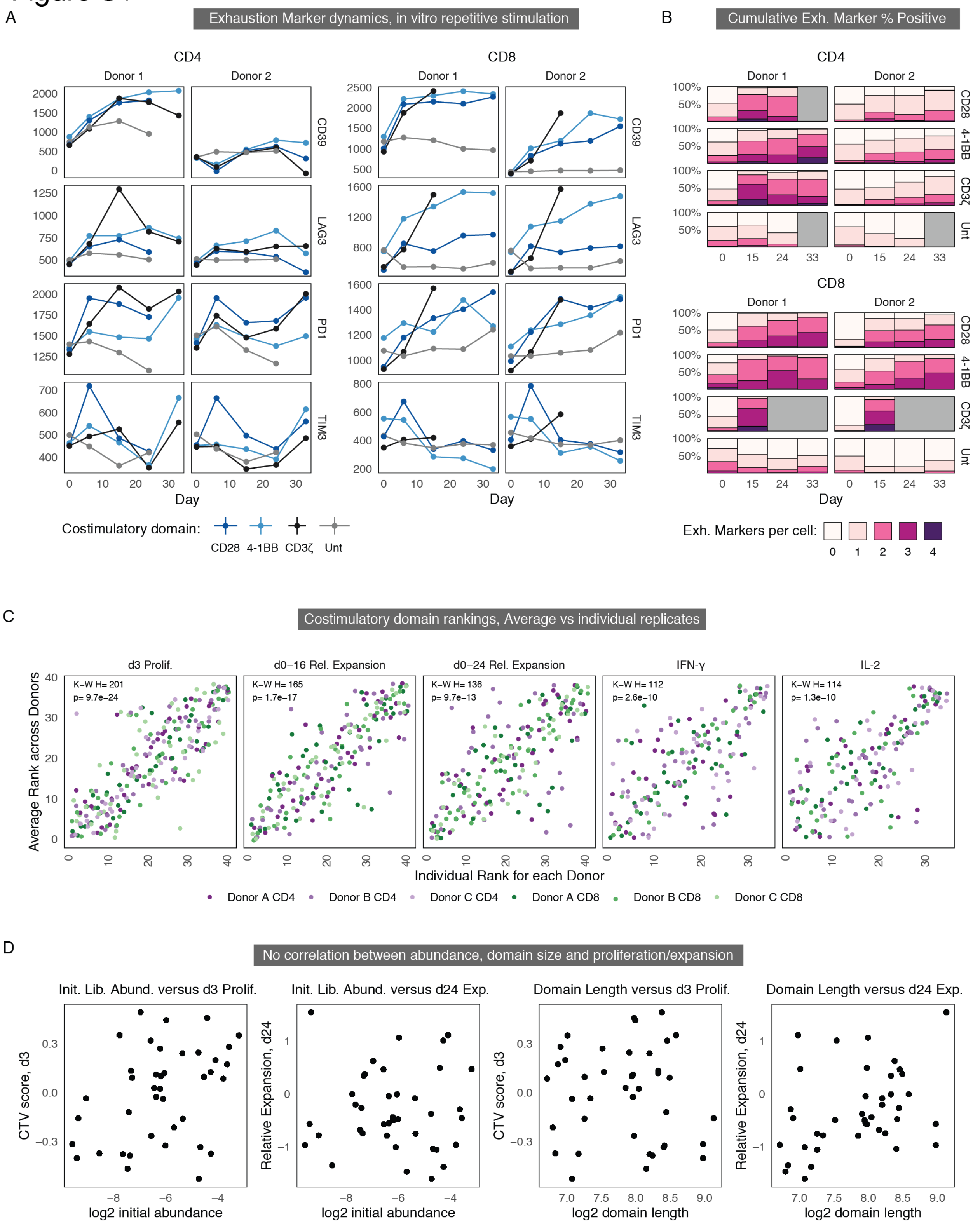

A
Exhaustion Marker dynamics, in vitro repetitive stimulation
CD4
CD8
Donor 1
Donor 2
CD39
LAG3
PD1
TIM3
Day
Costimulatory domain:
CD28
4-1BB
CD3ζ
Unt
B
Cumulative Exh. Marker % Positive
CD4
CD8
Exh. Markers per cell:
C
Costimulatory domain rankings, Average vs individual replicates
d3 Prolif.
d0–16 Rel. Expansion
d0–24 Rel. Expansion
IFN-γ
IL-2
K–W H= 201 p= 9.7e–24
K–W H= 165 p= 1.7e–17
K–W H= 136 p= 9.7e–13
K–W H= 112 p= 2.6e–10
K–W H= 114 p= 1.3e–10
Average Rank across Donors
Individual Rank for each Donor
Donor A CD4
Donor B CD4
Donor C CD4
Donor A CD8
Donor B CD8
Donor C CD8
D
No correlation between abundance, domain size and proliferation/expansion
Init. Lib. Abund. versus d3 Prolif.
Init. Lib. Abund. versus d24 Exp.
Domain Length versus d3 Prolif.
Domain Length versus d24 Exp.
CTV score, d3
Relative Expansion, d24
log2 initial abundance
log2 domain length

1

Fig. S1. Repetitive stimulation reproducibly induces exhaustion.

(A) Mean fluorescence intensity (MFI) for four cell surface markers of exhaustion (CD39, lymphocyte activating protein 3 (LAG3), programmed cell death protein 1 (PD1), T cell immunoglobulin domain and mucin domain 3 (TIM3)) in anti-CD19 chimeric antigen receptor (CAR) T cells generated from two donors measured after repeated stimulation with CD19-expressing irradiated K562 cells. CARs contained either 4-1BB or CD28 costimulation domains, no costimulatory domain (CD3ζ only), or were untransduced (Unt) T cells from the same donor as a control. **(B)** Aggregated measurements for panel (A) are shown, displayed as the percentage of cells expressing 0 to 4 exhaustion (Exh.) markers, for each donor, timepoint, and CAR. **(C)** Rank ordering of all 40 CARs within multiple assays is shown, with average rank for donor as well as CD4 and CD8 replicates plotted against the rank within the individual donors and T cell subsets. Proliferation (Prolif.), relative (Rel.) expansion, interferon (IFN)-γ production, and interleukin (IL)-2 production are shown. A Kruskal Wallis H test was performed for each assay, and the H statistic and p-value are shown. The upper right and lower left (lowest- and highest-ranked domains) are the most consistently ranked among individual replicates. **(D)** Initial library abundance (Init. Lib. Abund.) of each CAR (as a log2 fraction of the total read count) and domain length (log2 of the number of nucleotides) plotted versus either the Cell Trace Violet (CTV) score on day 3 (representing early proliferation) or the long-term expansion, log2-transformed and normalized to the average library member. None of these plots show any statistically significant correlation between either initial library abundance or costimulatory domain size and domain performance.

Figure S2

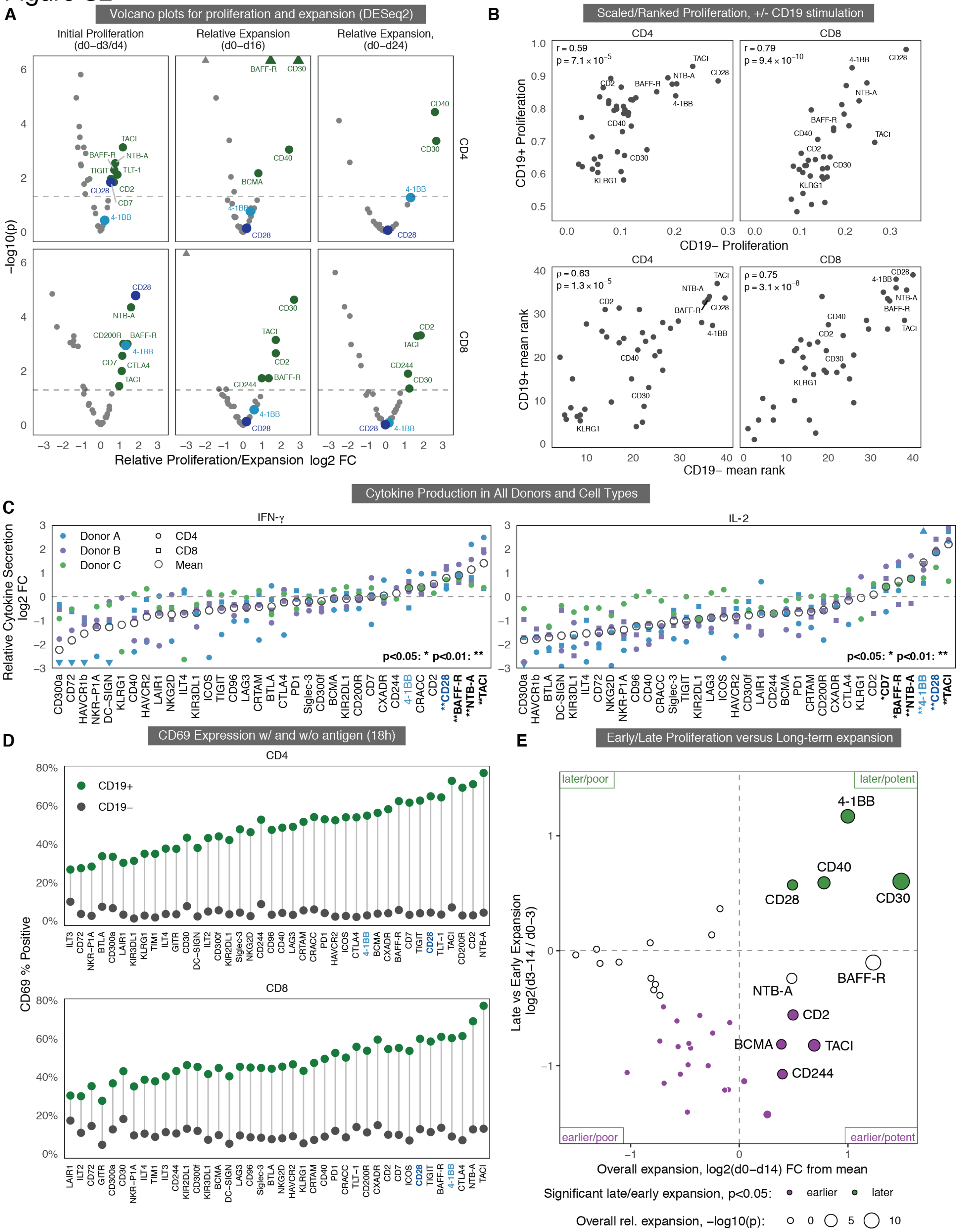

A
Volcano plots for proliferation and expansion (DESeq2)
Initial Proliferation (d0–d3/d4)
Relative Expansion (d0–d16)
Relative Expansion, (d0–d24)
–log10(p)
Relative Proliferation/Expansion log2 FC
CD4
CD8
B
Scaled/Ranked Proliferation, +/- CD19 stimulation
CD19+ Proliferation
CD19– Proliferation
CD19+ mean rank
CD19– mean rank
C
Cytokine Production in All Donors and Cell Types
IFN-γ
IL-2
Relative Cytokine Secretion log2 FC
Donor A
Donor B
Donor C
CD4
CD8
Mean
p<0.05: * p<0.01: **
D
CD69 Expression w/ and w/o antigen (18h)
CD69 % Positive
CD19+
CD19–
E
Early/Late Proliferation versus Long-term expansion
later/poor
later/potent
earlier/poor
earlier/potent
Late vs Early Expansion log2(d3–14 / d0–3)
Overall expansion, log2(d0–d14) FC from mean
Significant late/early expansion, p<0.05: earlier later
Overall rel. expansion, –log10(p): 0 5 10

1

Fig. S2. Additional data and statistical correlations for the differential activation, proliferation, and long-expansion in a library of CAR-T costimulatory domain variants. **(A)** Volcano plots showing the relative proliferation or expansion (according to panel labels) of CD4 or CD8 T cells expressing CARs containing different costimulatory domains, during the repetitive stimulation assay with CD19+ K562 cells. The x-axis shows the calculated difference in log2-fold change (FC) in proliferation or expansion, and the y-axis shows the associated adjusted P-value, as calculated by the DESeq2 algorithm. BAFF-R, B cell activating factor receptor; TACI, Transmembrane activator calcium modulator and cyclophilin ligand interactor; TIGIT, T cell immunoreceptor with Ig and ITIM domains; NTB-A, NK-T-B-antigen; TLT-1, Triggering receptors expressed on myeloid cells-like transcript-1; BCMA, B-cell maturation antigen. **(B)** A comparison of CAR T cell proliferation from d0-d3 across the library with and without CD19 stimulation. The top plots show the scaled proliferation averaged over each replicate but retain the differences in relative proliferation between CD19- and CD19+ conditions, which were measured simultaneously in our FlowSeq CTV assay. The bottom plots show the mean CAR rankings separately for the CD19- and CD19+ conditions. The top-performing potent costimulatory CARs are labeled. On the top, the y-axis is truncated due to the higher relative proliferation in the CD19+ condition. **(C)** FlowSeq measurement of intracellular cytokine production are shown across library domains in CD4 and CD8 (circle, square) T cells across three independent human donors (blue, purple, green), 18 hours after the initial addition of CD19+/- irradiated K562 cells. Means of all conditions for each cytokine are indicated by an open circle. Domains labeled in bold with stars next to their name indicate significance using a Wilcoxon rank-sum test, FDR-corrected $p < 0.05$. HAVCR, Hepatitis A virus cellular receptor 1; NKR-P1A, natural killer cell surface protein P1A; KLRG1, killer cell lectin like receptor G1; LAIR1, leukocyte associated immunoglobulin like receptor 1; ILT4, immunoglobulin-like transcript 4; KIR, killer cell immunoglobulin-like receptor; ICOS, inducible T cell costimulator; CRTAM, cytotoxic and regulatory T cell molecule; BTLA, B and T lymphocyte attenuator; CTLA4, cytotoxic T-lymphocyte associated protein 4; CXADR, Coxsackie virus and adenovirus receptor; CRACC, CD2-like receptor activating cytotoxic cells. **(D)** FlowSeq measurement of the percentage of CD69+ cells is shown for each CAR library domain in both CD4 and CD8 cells, 18 hours after the addition of irradiated K562 cells either with or without CD19 expression. Cells are ranked based on the difference in percentage of CD69+ cells between CD19+ and CD19- conditions. **(E)** A comparison of early versus late antigen-

stimulated proliferation is shown. The x- axis measures overall expansion by day 14 or 16 (d14/16) with more potent CARs on the right and less potent CARs on the left. The y-axis measures the ratio of late proliferation (d3 to d14) versus early proliferation (d0 to d3). CARs above 0 on the y-axis are more expanded in the library at later time points, and CARs below 0 are more expanded earlier. Domains significantly enriched earlier versus later during the expansion were colored purple and green, respectively. Significance of each domain's overall relative expansion indicated by size of circle (Wald test using DeSEQ2, -log10(p)).

Figure S3

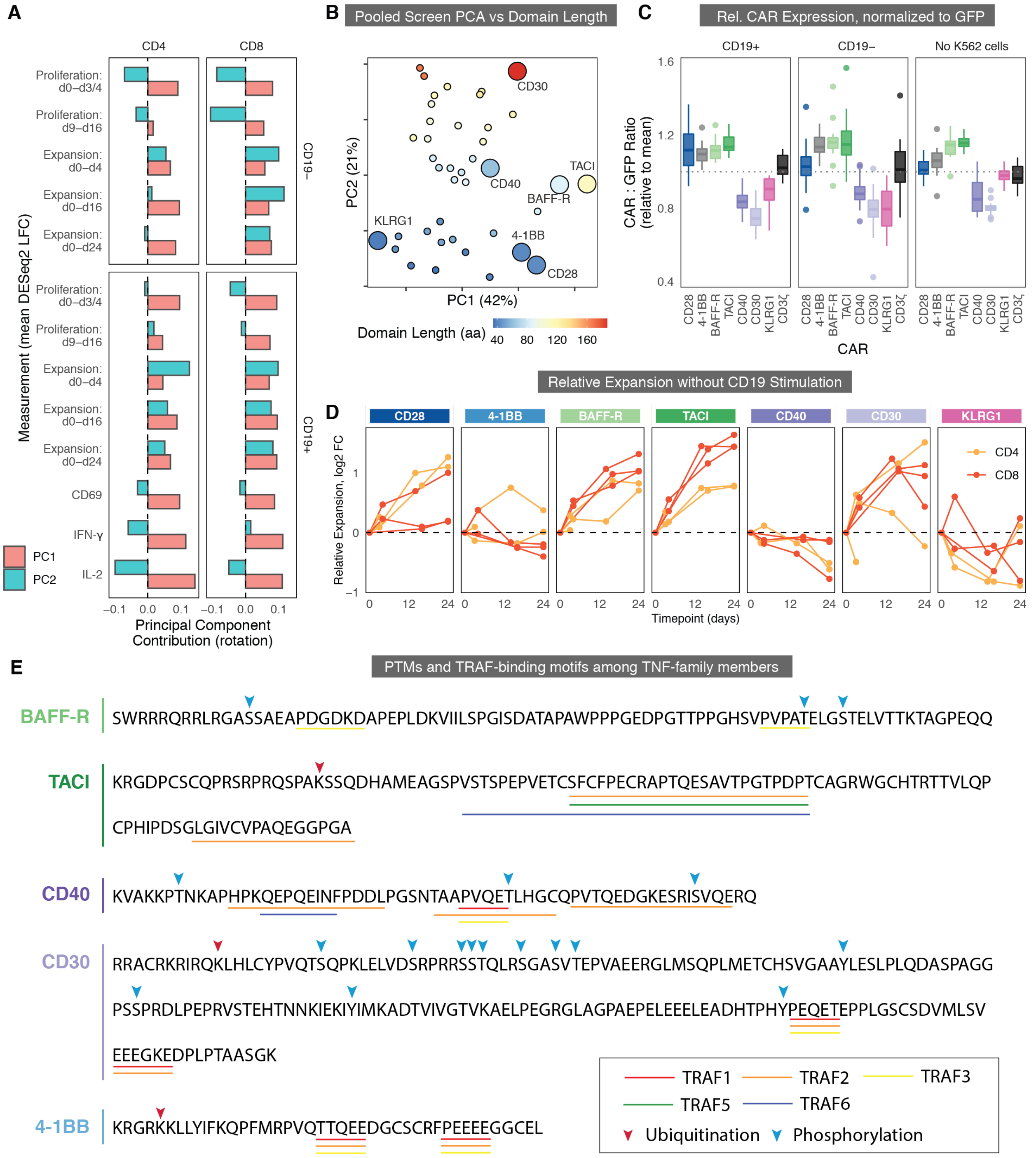
A
CD4
CD8
Proliferation: d0–d3/4
Proliferation: d9–d16
Expansion: d0–d4
Expansion: d0–d16
Expansion: d0–d24
CD69
IFN-γ
IL-2
CD19–
CD19+
Measurement (mean DESeq2 LFC)
PC1
PC2
Principal Component Contribution (rotation)
B
Pooled Screen PCA vs Domain Length
PC2 (21%)
PC1 (42%)
CD30
CD40
TACI
BAFF-R
KLRG1
4-1BB
CD28
Domain Length (aa)
40 80 120 160
C
Rel. CAR Expression, normalized to GFP
CD19+
CD19–
No K562 cells
CAR : GFP Ratio (relative to mean)
CAR
CD28 4-1BB BAFF-R TACI CD40 CD30 KLRG1 CD3ζ
D
Relative Expansion without CD19 Stimulation
CD28
4-1BB
BAFF-R
TACI
CD40
CD30
KLRG1
CD4
CD8
Relative Expansion, log2 FC
Timepoint (days)
E
PTMs and TRAF-binding motifs among TNF-family members
BAFF-R
SWRRRQRRLRGASSAEAPDGDKDAPEPLDKVIILSPGISDATAPAWPPPGEDPGTTPPGHSVPVPATELGSTELVTTKTAGPEQQ
TACI
KRGDPCSCQPRSRPRQSPAKSSQDHAMEAGSPVSTSPEPVETCSFCFPECRAPTQESAVTPGTPDPTCAGRWGCHTRTTVLQP
CPHIPDSGLGIVCVPAQEGGPGA
CD40
KVAKKPTNKAPHPKQEPQEINFPDDLPGSNTAAPVQETLHGCQPVTQEDGKESRISVQERQ
CD30
RRACRKRIRQKLHLCYPVQTSQPKLELVDSRPRRSSTQLRSGASVTEPVAEERGLMSQPLMETCHSVGAAYLESLPLQDASPAGG
PSSPRDLPEPRVSTEHTNNKIEKIYIMKADTVIVGTVKAELPEGRGLAGPAEPELEEELEADHTPHYPEQETEPPLGSCSDVMLSV
EEEGKEDPLPTAASGK
4-1BB
KRGRKKLLYIFKQPFMRPVQTTQEEDGCSCRFPEEEEGGCEL
TRAF1
TRAF2
TRAF3
TRAF5
TRAF6
Ubiquitination
Phosphorylation

Fig. S3. Functional characterization of costimulatory landscape and analysis of the composition of the principal components of CAR performance across our library.

(A) The bar plots show the relative contributions of different measurement types (in CD4 T cells and CD8 T cells, with and without antigenic stimulation) to each principal component (PC) of the PC analysis (PCA) plot in Fig. 3B and fig. S3B. The y-axis indicates the mean log fold change (LFC) and the x-axis indicates the contribution of each PC. Contributions are grouped across donor replicates and separated out by different timepoints, proliferation (cell trace violet (CTV) FlowSeq), expansion (change in relative library abundance over time), intracellular cytokine FlowSeq, and activation (CD69 FlowSeq). PC1 (red) describes most of the variability in antigen-positive proliferation and expansion, and contributions to PC2 (blue) include early expansion (but not CTV-measured cell divisions), decreased CD4 cytokine secretion and reduced tonic signaling. **(B)** A recoloring of Fig. 3B is shown according to the amino acid length of each costimulatory domain, showing a slight correlation between domain length (blue to red is shortest to longest) and the second principal component, but not the first. **(C)** Ratio of surface CAR expression (using a myc tag and flow cytometry staining) to green fluorescent protein (GFP) fluorescence is shown for each CAR. All CAR variants were normalized to the mean within each time point, donor, and T cell type (CD4 or CD8). Expression with CD19+ K562 cells, CD19- K562 cells, and no target cells are shown separately. Box and whisker plots indicate median CAR:GFP ratio and variance as plotted by interquartile range, minimum, and maximum (excluding outliers plotted separately) for each measured CAR. **(D)** Relative expansion of library members CD28, 4-1BB, BAFF-R, TACI, CD40, CD30, and KLRG1 is shown over 24 days of repeated stimulation with irradiated CD19- K562 cells, as in Fig. 3C. Expansion was quantified by calculating the fold-change of the proportion of each CAR within the library at each timepoint (x-axis) as compared to baseline relative to the average CAR within the pooled library. The library was measured in CD4 and CD8 primary human T cells individually in 2 to 3 biological replicates. **(E)** Amino acid sequence and motif analysis of selected library members' belonging to the tumor necrosis factor (TNF) receptor family. TNF receptor associated factor (TRAF) binding sites indicated with colored lines under amino acid sequence. Phosphorylation and ubiquitination sites as annotated by Phosphosite are indicated with blue and red downward arrows, respectively.

Figure S4

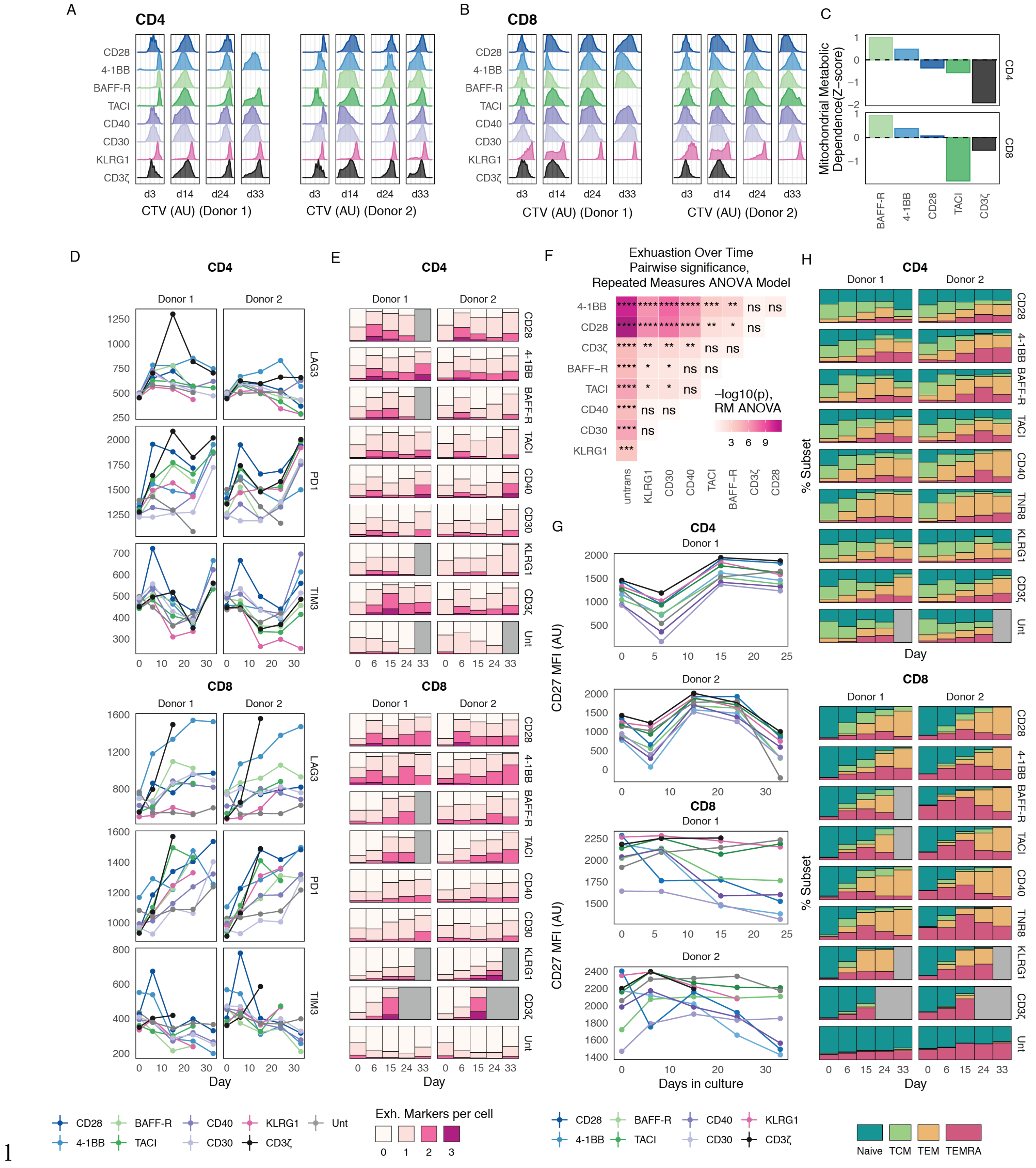
A
CD4
CD28
4-1BB
BAFF-R
TACI
CD40
CD30
KLRG1
CD3ζ
d3 d14 d24 d33
CTV (AU) (Donor 1)
CTV (AU) (Donor 2)
B
CD8
C
Mitochondrial Metabolic Dependence(Z-score)
BAFF-R 4-1BB CD28 TACI CD3ζ
D
Donor 1
Donor 2
LAG3
PD1
TIM3
Day
E
Exh. Markers per cell
0 1 2 3
Unt
F
Exhuastion Over Time
Pairwise significance,
Repeated Measures ANOVA Model
untrans
–log10(p), RM ANOVA
G
CD27 MFI (AU)
Days in culture
H
% Subset
TNR8
Naive TCM TEM TEMRA
CD28 4-1BB BAFF-R TACI CD40 CD30 KLRG1 CD3ζ Unt

Fig. S4. Proliferation, exhaustion, and differentiation characteristics of CARs with chosen costimulatory domains, and metabolism of CARs with chosen costimulatory domains.

(A) CTV flow cytometry histograms are shown, as in Fig. 4C, for both donors, all time points, and CD4 T cells. AU, arbitrary units. **(B)** CTV cytometry histograms are shown, as in Fig. 4C, for both donors, all time points and CD8 T cells. **(C)** Normalized relative metabolic mitochondrial dependence for CD4 and CD8 T cells was measured among select CARs. This metric is based on measurement of protein synthesis using the simple method for complex immune-metabolic profiling (SCENITH), which calculates the change in overall metabolic output with and without the addition of oligomycin, a mitochondrial inhibitor. **(D)** MFI for three cell surface markers of exhaustion (LAG3, PD1, and TIM3) is shown for anti-CD19 CAR T cells generated from two donors, measured after repeated stimulation with CD19+ irradiated K562 cells. **(E)** The proportion of CAR T cells expressing 0 to 3 of the exhaustion (Exh.) markers PD1, TIM3, and LAG3 after different numbers of days in culture is shown, as in Fig. 4F. **(F)** A table of significant differences in pairwise statistical tests based on a Repeated Measures ANOVA model is shown for mean exhaustion markers across different subtypes, donors, and days of measurement. FDR < 0.05:*, < 0.01:**, < 0.001:***, < 0.0001:****; ns, not significant. **(G)** MFI of CD27 was measured across all T cells, timepoints, and CAR T variants, as in Fig. 4G**. (H)** Differentiation of T cells at different timepoints throughout the repeated stimulation assay was evaluated. Differentiation subsets [Naive, Central Memory (TCM), Effector Memory (TEM), and Effector Memory RA-positive (TEMRA)] were calculated using surface expression of CD45RA and CD62L,.

Figure S5

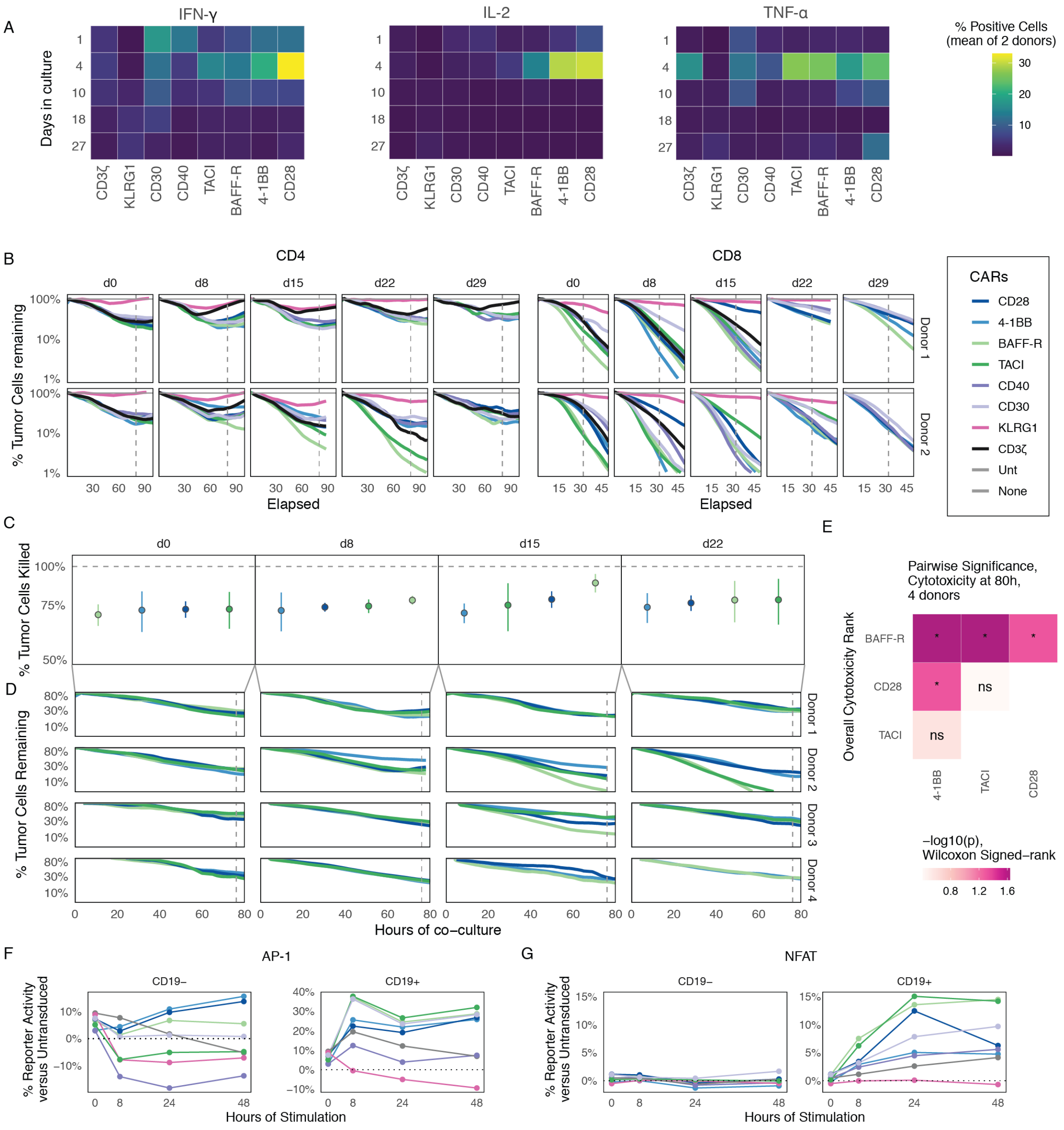
A
IFN-γ
IL-2
TNF-α
Days in culture
1
4
10
18
27
CD3ζ
KLRG1
CD30
CD40
TACI
BAFF-R
4-1BB
CD28
% Positive Cells
(mean of 2 donors)
30
20
10
B
CD4
CD8
d0
d8
d15
d22
d29
% Tumor Cells remaining
100%
10%
1%
Donor 1
Donor 2
Elapsed
CARs
CD28
4-1BB
BAFF-R
TACI
CD40
CD30
KLRG1
CD3ζ
Unt
None
C
% Tumor Cells Killed
100%
75%
50%
D
% Tumor Cells Remaining
80%
30%
10%
Donor 3
Donor 4
Hours of co−culture
E
Pairwise Significance,
Cytotoxicity at 80h,
4 donors
Overall Cytotoxicity Rank
BAFF-R
CD28
TACI
4-1BB
TACI
CD28
*
ns
−log10(p),
Wilcoxon Signed−rank
0.8
1.2
1.6
F
AP-1
CD19−
CD19+
% Reporter Activity
versus Untransduced
Hours of Stimulation
G
NFAT

Fig. S5. Time course of cytokine production, cytotoxicity, and transcriptional activity across CARs with chosen costimulatory domains.

(A) Mean cytokine production is shown across all T cells, time points, and CAR T variants, as in Fig. 5B. **(B)** All cytotoxicity plots across both CD4 and CD8 donors and all measured days are shown as in Fig 5E. **(C)** Cytotoxicity of CAR T cells were quantified at 80 hours for all four CD4 T cell donors (left) or at 32 hours for all CD8 T cell donors (right) expressing BAFF-R, TACI, CD28, or 4-1BB as in Fig. 5. Colors for each CAR are indicated in the legend. CARs are ranked at each timepoint from least to most cytotoxic (left to right). **(D)** Representative plots of cytotoxicity of CD4 CAR T cells from all four donors expressing BAFF-R, TACI, CD28, or 4-1BB are shown, with colors labeled as in (B). CARs are ranked at each timepoint from least to most cytotoxic (left to right). Vertical dashed lines indicate the time points analyzed in (C). Error bars indicate the standard error calculated across donors. **(E)** Table of significant differences in CD4 cytotoxicity shown in (D) using pairwise statistical tests across the chosen 4 donors and 4 CARs. Significance scores are based on a Repeated Measures ANOVA model of percentage of cell killing at 80 hours across different donors and days of repetitive stimulation (FDR < 0.05:*; ns, not significant). **(F)** Transcriptional activity reporter Jurkat cell lines for activator protein 1 (AP-1) were transduced with each CAR and sorted within one log of GFP expression. The cells were stimulated with either CD19- or CD19+ K562 cells for 0, 8, 24, or 48 hours and then assessed for activity by flow cytometry. Percent transcription factor activity relative to untransduced reporter Jurkat cells is plotted on the y-axis. **(G)** Transcriptional activity reporter Jurkat cell lines for nuclear factor of activated T-cells (NFAT) as described in (F).

Figure S6

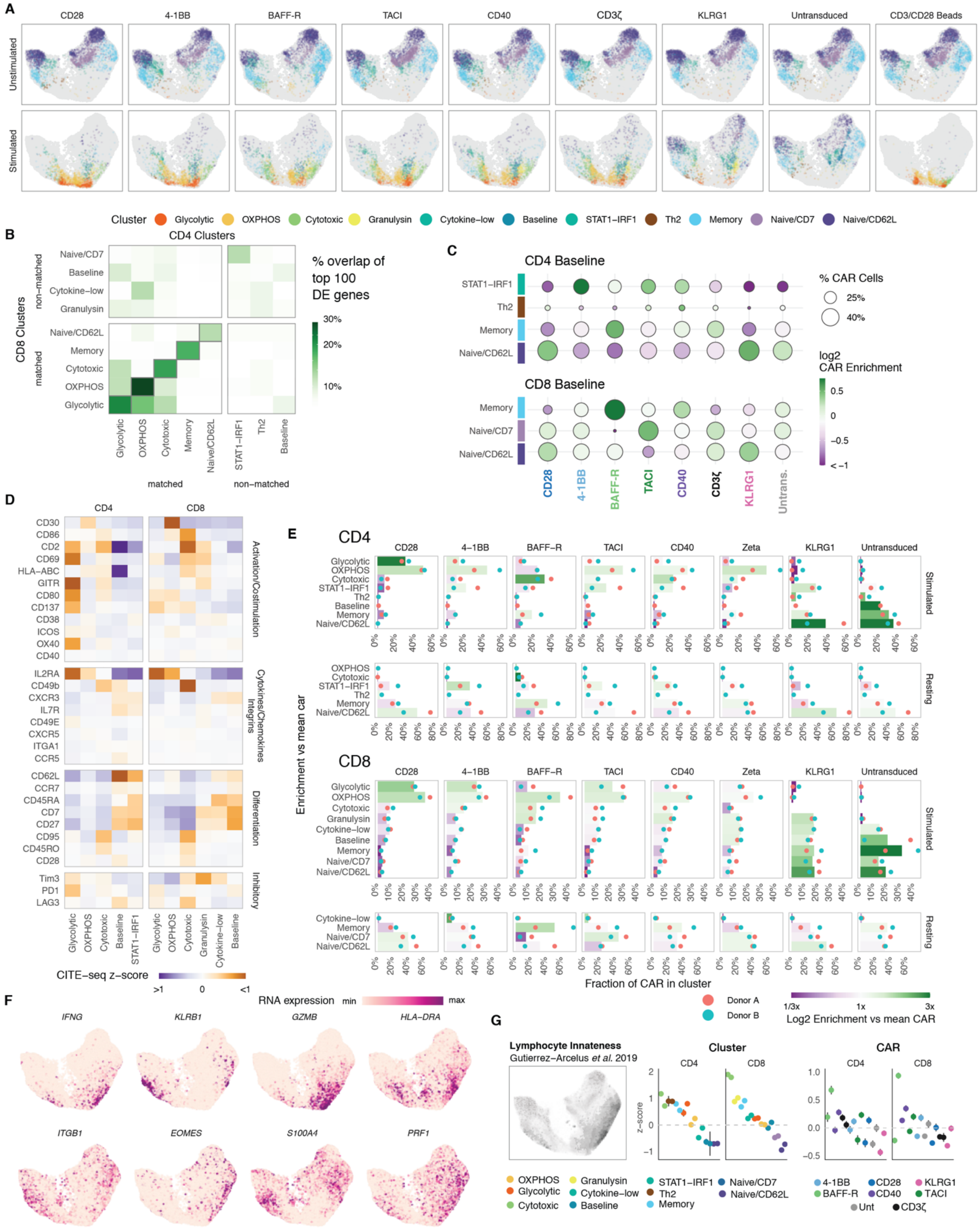
A
CD28
4-1BB
BAFF-R
TACI
CD40
CD3ζ
KLRG1
Untransduced
CD3/CD28 Beads
Unstimulated
Stimulated
Cluster
Glycolytic
OXPHOS
Cytotoxic
Granulysin
Cytokine–low
Baseline
STAT1–IRF1
Th2
Memory
Naive/CD7
Naive/CD62L
B
CD4 Clusters
CD8 Clusters
non–matched
matched
% overlap of top 100 DE genes
30%
20%
10%
C
CD4 Baseline
CD8 Baseline
% CAR Cells
25%
40%
log2 CAR Enrichment
0.5
0
−0.5
< −1
Untrans.
D
CD4
CD8
CD30
CD86
CD2
CD69
HLA-ABC
GITR
CD80
CD137
CD38
ICOS
OX40
CD40
Activation/Costimulation
IL2RA
CD49b
CXCR3
IL7R
CD49E
CXCR5
ITGA1
CCR5
Cytokines/Chemokines Integrins
CD62L
CCR7
CD45RA
CD7
CD27
CD95
CD45RO
CD28
Differentiation
Tim3
PD1
LAG3
Inhibitory
CITE–seq z–score
>1
0
<1
E
CD4
CD8
Zeta
Stimulated
Resting
Enrichment vs mean car
Fraction of CAR in cluster
Donor A
Donor B
1/3x
1x
3x
Log2 Enrichment vs mean CAR
F
RNA expression
min
max
IFNG
KLRB1
GZMB
HLA-DRA
ITGB1
EOMES
S100A4
PRF1
G
Lymphocyte Innateness
Gutierrez–Arcelus et al. 2019
Cluster
CAR
z–score
Naive/CD7
Naive/CD62L
4-1BB
BAFF-R
CD28
CD40
KLRG1
TACI
Unt
CD3ζ

Fig. S6. Single-cell analysis of CARs with chosen costimulatory domains with and without antigen stimulation.

(A) Uniform manifold approximation and projection (UMAP) plots are faceted separately for each CAR costimulatory domain and stimulation condition. Points are colored the same as Fig. 6A. An additional CD3/CD28 bead stimulation condition is also shown, which was done only in Donor 2. **(B)** Gene expression overlap is shown across 5 pairs of clusters, which are very similar between CD4 and CD8 T cells (Naive/CD62L, Memory, Cytotoxic, OXPHOS, and Glycolytic). A list of the top 100 differentially expressed genes was calculated for each cluster among all CD4 or CD8 T cells. This plot shows the percentage overlap in these gene lists between clusters, showing a mirroring of gene expression across the CD4-CD8 axis among the 5 matched clusters in the bottom left quadrant. **(C)** Enrichment of resting CAR T cells containing different signaling domains within each phenotypic cluster, similar to Fig. 6E. The size of each dot corresponds to the percentage of stimulated CAR T cells with a specific costimulatory domain that is assigned to a cluster. The color of each dot corresponds to the log-2 fold enrichment or depletion of that CAR within the cluster. **(D)** Cellular indexing of transcriptomes and epitopes by sequencing (CITE-seq) z-scores are shown for a variety of surface proteins among T cells in different activated clusters, grouped by their functional classification. Z-scores for CD4 and CD8 T cells were calculated separately. **(E)** A breakdown of the cluster frequency is shown among all stimulated and resting CAR variants of both donors. The bar length on the x-axis is the percentage of each costimulatory CAR variant (resting and stimulated separately) within that cluster, such that each set of bars within each faceted box sums to 1. The bars represent the mean percentage for both donors, and the blue and red dots represent the individual percentages for each donor. The color of each bar corresponds to the relative log2 enrichment for that CAR variant in that cluster, relative to other CAR variants. **(F)** UMAP heatmaps display the relative RNA expression of single cells (scaled individually), showing a subset of functionally-important transcripts that are upregulated in the Cytotoxic and Memory subsets. **(G)** Correlation of T cell gene signatures indicative of lymphocyte innateness, based on Gutierrez-Arcelus *et al.* (64) (left) with phenotypic clusters in CD4 and CD8 CAR T cells (middle) or with CARs containing different costimulatory domains (right). Cluster and CAR colors match those in Fig. 6F. The two dots per group correspond to donors A and B. Error bars indicate 99% confidence intervals for the z-scores.

Figure S7

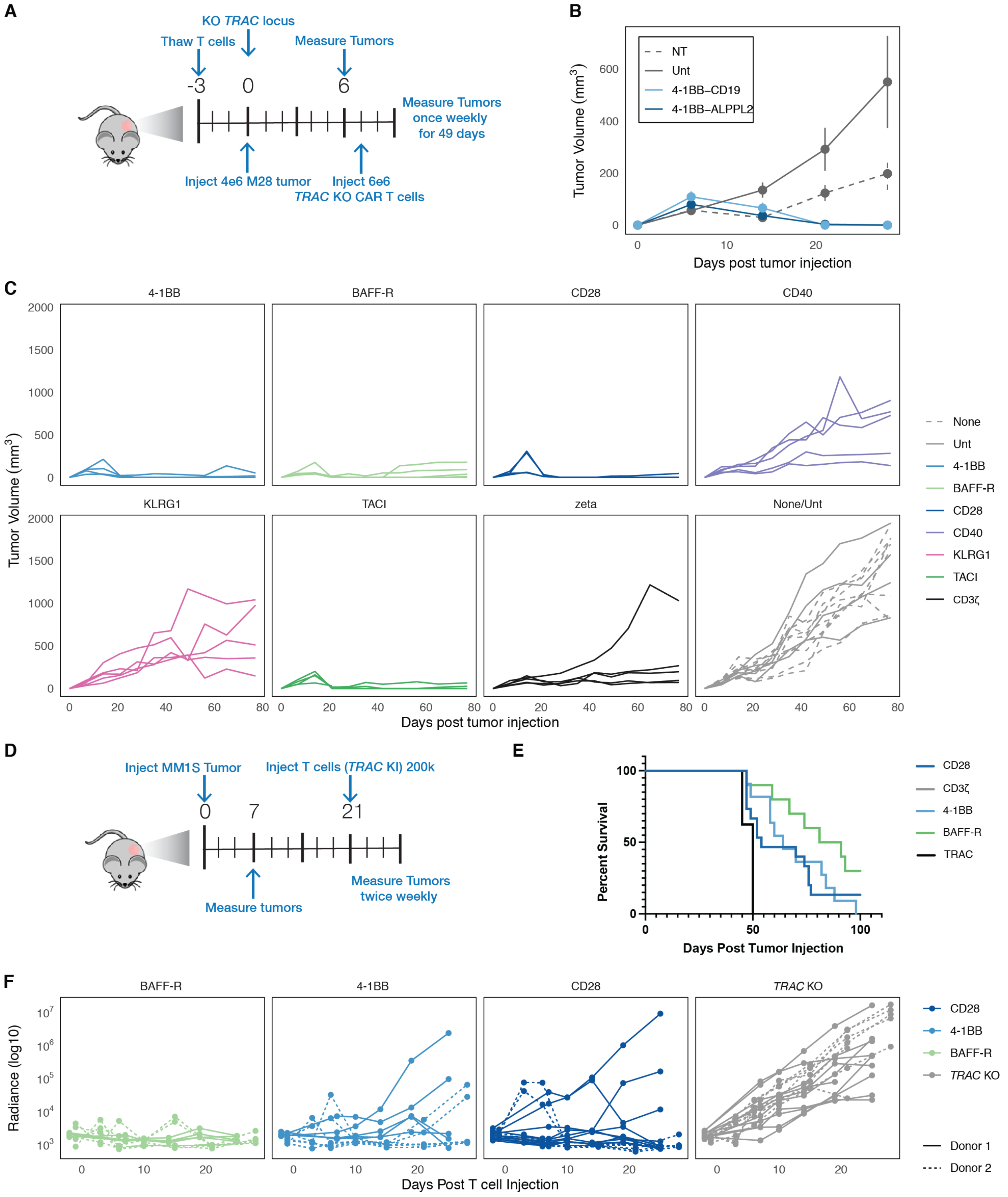

A
KO TRAC locus
Thaw T cells
Measure Tumors
-3
0
6
Measure Tumors once weekly for 49 days
Inject 4e6 M28 tumor
Inject 6e6 TRAC KO CAR T cells
B
NT
Unt
4-1BB–CD19
4-1BB–ALPPL2
Tumor Volume (mm3)
Days post tumor injection
C
4-1BB
BAFF-R
CD28
CD40
KLRG1
TACI
zeta
None/Unt
Tumor Volume (mm3)
Days post tumor injection
None
Unt
4-1BB
BAFF-R
CD28
CD40
KLRG1
TACI
CD3ζ
D
Inject MM1S Tumor
Inject T cells (TRAC KI) 200k
0
7
21
Measure tumors
Measure Tumors twice weekly
E
Percent Survival
Days Post Tumor Injection
CD28
CD3ζ
4-1BB
BAFF-R
TRAC
F
BAFF-R
4-1BB
CD28
TRAC KO
Radiance (log10)
Days Post T cell Injection
CD28
4-1BB
BAFF-R
TRAC KO
Donor 1
Donor 2

1

Fig S7. In vivo efficacy of highlighted signaling domains in the M28 and MM1S cancer models.

(A) Experimental timeline for in vivo M28 mesothelioma tumor model. We injected 4x10^6 CD19+ M28 mesothelioma tumor cells subcutaneously into the flanks of NOD.Cg-Prkdc scid Il2rg tm1Wjl /SzJ (NSG) mice and, seven days later, transferred 6x10^6 engineered *TRAC* knockout (KO) CAR T cells targeting CD19 intravenously into the tail vein. Tumors were measured by caliper every 7 days for a total of 49 days. **(B)** Tumor burden was measured in mice treated with CAR T cells targeting either ALPPL2 or CD19. Untransduced (Unt) T cells and non-treated (NT) mice were included as controls. Tumors were measured by caliper every 7 days for a total of 30 days. Error bars indicate standard error of the mean for tumor volumes across mice. **(C)** M28 tumor volume was plotted over time for individual mice, corresponding to the mean tumor volumes in Fig. 7A and B. **(D)** Experimental timeline for in vivo MM1S multiple myeloma tumor model is shown. We injected 1x10^6 MM1S multiple myeloma tumor cells intravenously into NSG mice and, three weeks later, transferred 200,000 engineered *TRAC*-knockin CAR T cells targeting BCMA intravenously into the tail vein. **(E)** Survival curves are shown for mice treated with CAR T cells derived from Donor 1 and Donor 2 in the MM1S tumor model; results were combined for both donors. Mice were monitored over 100 days. **(F)** MM1S tumor volume is shown plotted over time for individual mice in all treatments, corresponding to Fig. 7C.

Table S1. Expression of individual signaling domains by receptor type.

The table shows a list of all costimulatory domains in our library and whether they are expressed by different immune cell types. Note that some receptors may have low expression or may only be expressed under specific circumstances by individual cell types.

Supplemental Table 1. Expression of individual cosignaling receptors by cell type

Cosignaling Receptor	T cel	B cel	NK cel	DC C	Macrophag	NKT C	Granulocy	Microgl	Total Cell Types
41BB	X								1
BAFF-R	X	X							2
BCMA		X							1
BTLA	X	X							2
CD2	X		X						2
CD200R	X						X		2
CD244	X		X				X		3
CD28	X	X					X		3
CD300a	X	X	X	X			X		5
CD300f									0
CD40		X			X				2
CD7	X		X						2
CD72		X							1
CD96	X		X						2
CRACC	X	X	X						3
CRTAM	X					X			2
CTLA4	X	X					X		3
CXADR									0
DC-SIGN				X					1
GITR	X		X						2
TIM3	X		X	X					3
ICOS	X								1
ILT2		X	X	X	X				4
ILT3		X	X	X	X				4
ILT4		X	X	X					3
KIR2DL1			X						1
KIR3DL1	X		X						2
KLRG1	X		X						2
LAG3	X		X						2
LAIR1	X	X	X	X					4
NKG2D	X		X	X					3
NKR-P1A	X		X						2
NTB-A	X	X	X	X					4
PD1	X	X	X	X	X			X	6
Siglec-3				X				X	2
TACI	X	X							2
TIGIT	X		X						2
TLT-1									0
CD30	X	X	X		X				4

Table S2. List of reagents used in this study.

**Reagent**	**Source**	**Catalog Number**
Antibodies		
Monoclonal anti-human CD197(CCR7)-PE/Cy7 (clone G043H7)	BioLegend	Cat# 353226, RRID: AB_11126145
Monoclonal anti-human CD223(LAG-3)-AF647 (clone 11C3C65)	BioLegend	Cat# 369304, RRID: AB_2566480
Monoclonal anti-human CD27-APC/Cyanine7 (clone M-T271)	BioLegend	Cat# 356424, RRID: AB_2566773
Monoclonal anti-human CD297(PD-1)-BV711 (clone EH12.2H7)	BioLegend	Cat# 329928, RRID: AB_2562911
Monoclonal anti-human CD366(Tim-3)-BV421 (clone F38-2E2)	BioLegend	Cat# 345008, RRID: AB_11218598
Monoclonal anti-human CD39-APC/Cyanine7 (clone A1)	BioLegend	Cat# 328226, RRID: AB_2571981
Monoclonal anti-human CD4-PE (clone OKT4)	BioLegend	Cat# 317410, RRID: AB_571955
Monoclonal anti-human CD4-PE (clone SK3)	BioLegend	Cat# 344606, RRID: AB_1937246
Monoclonal anti-human CD4-BUV395 (clone SK3)	BD Biosciences	Cat# 563552
Monoclonal anti-human CD4-Pacific Blue (clone SK3)	BioLegend	Cat# 344620, RRID: AB_2228841
Monoclonal anti-human CD45RA-APC (clone HI100)	BioLegend	Cat# 304112, RRID: AB_314416
Monoclonal anti-human CD45RO-BUV395 (clone UCHL1)	BD Biosciences	Cat# 564291
Monoclonal anti-human CD62L-BV785 (clone DREG-56)	BioLegend	Cat# 304830, RRID: AB_2629555

Monoclonal anti-human CD8-PE (clone SK1)	BioLegend	Cat# 344706, RRID: AB_1953244
Monoclonal anti-human CD8-BUV395 (clone RPA-T8)	BD Biosciences	Cat# 563795
Monoclonal anti-human CD8-Pacific Blue (clone SK1)	BioLegend	Cat# 344718, RRID: AB_10551438
Monoclonal anti-human CD95-BV711 (clone DX2)	BioLegend	Cat# 305644, RRID: AB_2632623
Monoclonal anti-human c-Myc-AF594 (clone 9B11)	Cell Signaling Technology	Cat# 9483S
Monoclonal anti-human IFN-γ-BV786 (clone 4S.B3)	BD Biosciences	Cat# 563731
Monoclonal anti-human IL-2-APC (clone MQ1-17H12)	BD Biosciences	Cat# 554567
Monoclonal anti-human TNF-BUV395 (clone MAb11)	BD Biosciences	Cat# 563996
TotalSeq-A0251 HT1	BioLegend	Cat# 394601
TotalSeq-A0252 HT2	BioLegend	Cat# 394603
TotalSeq-A0253 HT3	BioLegend	Cat# 394605
TotalSeq-A0254 HT4	BioLegend	Cat# 394607
TotalSeq-A0255 HT5	BioLegend	Cat# 394609
TotalSeq-A0256 HT6	BioLegend	Cat# 394611
TotalSeq-A0257 HT7	BioLegend	Cat# 394613
TotalSeq-A0258 HT8	BioLegend	Cat# 394615
TotalSeq-A0259 HT9	BioLegend	Cat# 394617
TotalSeq-A0260 HT10	BioLegend	Cat# 394619
TotalSeq-A0262 HT12	BioLegend	Cat# 394623
TotalSeq-A0263 HT13	BioLegend	Cat# 394625
ABSeq Oligo Mouse Monoclonal anti-	BD	Cat# 940046

human CD2	Biosciences	
ABSeq Oligo Mouse Monoclonal anti-human CD3	BD Biosciences	Cat# 940000
ABSeq Oligo Mouse Monoclonal anti-human CD183	BD Biosciences	Cat# 940030
ABSeq Oligo Mouse Monoclonal anti-human CD103	BD Biosciences	Cat# 940067
ABSeq Oligo Mouse Monoclonal anti-human CD270	BD Biosciences	Cat# 940097
ABSeq Oligo Mouse Monoclonal anti-human CD54	BD Biosciences	Cat# 940072
ABSeq Oligo Mouse Monoclonal anti-human CD45RA	BD Biosciences	Cat# 940011
ABSeq Oligo Mouse Monoclonal anti-human CD197	BD Biosciences	Cat# 940014
ABSeq Oligo Mouse Monoclonal anti-human CD11a	BD Biosciences	Cat# 940077
ABSeq Oligo Mouse Monoclonal anti-human CD194	BD Biosciences	Cat# 940047
ABSeq Oligo Mouse Monoclonal anti-human CD336	BD Biosciences	Cat# 940085
ABSeq Oligo Mouse Monoclonal anti-human CD126	BD Biosciences	Cat# 940090
ABSeq Oligo Mouse Monoclonal anti-human CD123	BD Biosciences	Cat# 940020
ABSeq Oligo Mouse Monoclonal anti-human CD5	BD Biosciences	Cat# 940038
ABSeq Oligo Mouse Monoclonal anti-human CD196	BD Biosciences	Cat# 940033
ABSeq Oligo Mouse Monoclonal anti-	BD	Cat# 940089

human CD178	Biosciences	
ABSeq Oligo Mouse Monoclonal anti-human CD24	BD Biosciences	Cat# 940028
ABSeq Oligo Mouse Monoclonal anti-human CD56	BD Biosciences	Cat# 940007
ABSeq Oligo Mouse Monoclonal anti-human CD124	BD Biosciences	Cat# 940092
ABSeq Oligo Mouse Monoclonal anti-human CD185	BD Biosciences	Cat# 940042
ABSeq Oligo Mouse Monoclonal anti-human CD18	BD Biosciences	Cat# 940086
ABSeq Oligo Mouse Monoclonal anti-human IgG	BD Biosciences	Cat# 940027
ABSeq Oligo Mouse Monoclonal anti-human CD127	BD Biosciences	Cat# 940012
ABSeq Oligo Mouse Monoclonal anti-human CD25	BD Biosciences	Cat# 940009
ABSeq Oligo Mouse Monoclonal anti-human CD13	BD Biosciences	Cat# 940044
ABSeq Oligo Mouse Monoclonal anti-human CD1c	BD Biosciences	Cat# 940083
ABSeq Oligo Mouse Monoclonal anti-human CD278	BD Biosciences	Cat# 940043
ABSeq Oligo Mouse Monoclonal anti-human CD274	BD Biosciences	Cat# 940035
ABSeq Oligo Mouse Monoclonal anti-human CD11b	BD Biosciences	Cat# 940008
ABSeq Oligo Mouse Monoclonal anti-human CD49a	BD Biosciences	Cat# 940094
ABSeq Oligo Mouse Monoclonal anti-	BD	Cat# 940024

human CD11c	Biosciences	
ABSeq Oligo Mouse Monoclonal anti-human CD62L	BD Biosciences	Cat# 940041
ABSeq Oligo Mouse Monoclonal anti-human CD279	BD Biosciences	Cat# 940015
ABSeq Oligo Mouse Monoclonal anti-human CD195	BD Biosciences	Cat# 940050
ABSeq Oligo Mouse Monoclonal anti-human CD69	BD Biosciences	Cat# 940019
ABSeq Oligo Mouse Monoclonal anti-human CD335	BD Biosciences	Cat# 940064
ABSeq Oligo Mouse Monoclonal anti-human CD49b	BD Biosciences	Cat# 940087
ABSeq Oligo Mouse Monoclonal anti-human CD184	BD Biosciences	Cat# 940056
ABSeq Oligo Mouse Monoclonal anti-human CD30	BD Biosciences	Cat# 940103
ABSeq Oligo Mouse Monoclonal anti-human CD10	BD Biosciences	Cat# 940045
ABSeq Oligo Mouse Monoclonal anti-human CD223	BD Biosciences	Cat# 940080
ABSeq Oligo Mouse Monoclonal anti-human CD61	BD Biosciences	Cat# 940065
ABSeq Oligo Mouse Monoclonal anti-human IL-21R	BD Biosciences	Cat# 940099
ABSeq Oligo Mouse Monoclonal anti-human CD90	BD Biosciences	Cat# 940032
ABSeq Oligo Mouse Monoclonal anti-human CD80	BD Biosciences	Cat# 940036
ABSeq Oligo Mouse Monoclonal anti-	BD	Cat# 940081

human CD94	Biosciences	
ABSeq Oligo Mouse Monoclonal anti-human CD226	BD Biosciences	Cat# 940075
ABSeq Oligo Mouse Monoclonal anti-human HLA-ABC	BD Biosciences	Cat# 940062
ABSeq Oligo Mouse Monoclonal anti-human TCRgd	BD Biosciences	Cat# 940057
ABSeq Oligo Mouse Monoclonal anti-human CD86	BD Biosciences	Cat# 940025
ABSeq Oligo Mouse Monoclonal anti-human CD155	BD Biosciences	Cat# 940102
ABSeq Oligo Mouse Monoclonal anti-human CD206	BD Biosciences	Cat# 940068
ABSeq Oligo Mouse Monoclonal anti-human CD117	BD Biosciences	Cat# 940051
ABSeq Oligo Mouse Monoclonal anti-human CD95	BD Biosciences	Cat# 940037
ABSeq Oligo Mouse Monoclonal anti-human CD9	BD Biosciences	Cat# 940078
Bacterial and Virus Strains		
Escherichia coli: strain HST08 (Stellar Competent Cells)	Takara Bio	Cat# 636766
NEB 5-alpha Electrocompetent E. coli	New England Biosciences	Cat # C2989
Biological Samples		
T Cells from Donor D001004304	STEMCELL Technologies	Cat# 70500.2
T Cells from Donor RG1765	STEMCELL Technologies	Cat# 70500.1

T Cells from Donor RV01000251	STEMCELL Technologies	Cat# 70500.1
T Cells from Donor RG1310	STEMCELL Technologies	Cat# 70500.1
T Cells from Donor RG1945	STEMCELL Technologies	Cat# 70500.1
Chemicals, Peptides, and Recombinant Proteins		
Recombinant Human IL-2 Protein	R&D Systems	Cat# 202-IL-500
Acetic acid, glacial	Sigma-Aldrich	Cat# ARK2183-1L
CellTrace Violet	Thermo Fisher	Cat# C34557
eBioscience Brefeldin A Solution (1000X)	Invitrogen	Cat# 00-4506-51
Zombie Yellow Fixable Viability Kit	BioLegend	Cat# 423104
Dulbecco's Phosphate Buffered Saline	Sigma-Aldrich	Cat# D8537
X-VIVO 15	Lonza Bioscience	Cat# 04-418Q
Human AB Serum Heat Inactivated	Valley Biomedical, Inc	Cat# HP1022HI
N-Acetyl-L-Cysteine	Sigma-Aldrich	Cat# A9165
1.0N NaOH	Sigma-Aldrich	Cat# S2770
2-Mercaptoethanol	Gibco	Cat# 21985-023
RPMI 1640 Medium	Gibco	Cat# 11875-093
Glutamax	Fisher	Cat# 35050061

	Scientific	
Fetal Bovine Serum (Heat Inactivated)	SAFC Biosciences	Cat# 12306C-500ML
Penicillin-Streptomycin (10,000 IU/mL, 10,000 µg/mL)	MP Biomedicals	Cat# 1670249
InFusion	Takara Bio	Cat# 638951
EasySep Human CD4+ T Cell Isolation Kit	STEMCELL Technologies	Cat# 17952
EasySep Human CD8+ T Cell Isolation Kit	STEMCELL Technologies	Cat# 17953
EasySep Human T Cell Negative Isolation Kit	STEMCELL Technologies	Cat # 17951
Cyto-Last Buffer	BioLegend	Cat# 422501
NucleoSpin Tissue XS	Macherey-Nagel	Cat# 740901.50
NucleoSpin	Macherey-Nagel	Cat# 740952.50S
NucleoSpin 96 Tissue	Macherey-Nagel	Cat# 740741.4
TaKaRa Ex Taq DNA Polymerase	Takara Bio	Cat# RR001B
MiniSeq High Output Reagent Kit (150-cycles)	Illumina	Cat# FC-420-1002
HiSeq 4000 300 Cycle Kit	Illumina	Cat# FC-410-1003
eBioscience Intracellular Fixation & Permeabilization Buffer Set	Invitrogen	Cat# 88-8824-00
Experimental Models: Cell Lines		
Human: HEK293T	ATCC	
Human: K562 (CD19+mCherry+)	Lim Lab, UCSF	
Human: Nalm6	Eyquem Lab,	

(CD19+GFP+Luciferase+)	UCSF	
Human: M28	Gerwin Lab, NCI/NIH	
<u>*Experimental Models: Organisms/Strains*</u>		
Mouse: NOD.Cg-$Prkdc^{scid}$ $IL2rg^{tm1Wjl}$/Szj	The Jackson Laboratory	JAX: 005557
Software and Algorithms		
FlowJo version 10	FlowJo, LLC	https://www.flowjo.com
RStudio	RStudio	https://rstudio.com/
IncuCyte Base Software	Essen Bioscience (now part of Sartorius)	https://www.essenbioscience.com/en/
Living Image	PerkinElmer	https://www.perkinelmer.com
Prism version 9	Graph Pad	https://www.graphpad.com/scientific-software/prism/
<u>*Other*</u>		
Poly(A), Polyadenylic acid	Roche	Cat# 10108626001
Dynabeads Human T-Activator CD3/CD28	Thermo Fisher	Cat# 11131D
CountBright Absolute Counting Beads	Invitrogen	Cat# C36950
OneComp eBeads Compensation Beads	Invitrogen	Cat# 01-1111-42

Data file S1. T Cell Donor Demographic and Processing Information.

Data file S2. Raw, individual level data for experiments where n<20.
